# Metabolic engineering of *Yarrowia lipolytica* for the production and secretion of the saffron ingredient crocetin

**DOI:** 10.1186/s13068-024-02598-y

**Published:** 2025-01-07

**Authors:** Tingan Zhou, Young-Kyoung Park, Jing Fu, Piotr Hapeta, Cinzia Klemm, Rodrigo Ledesma-Amaro

**Affiliations:** 1 Department of Bioengineering, Imperial College Centre for Synthetic Biology, Bezos Centre for Sustainable Protein, UKRI Engineering Biology Mission Hub on Microbial Food, Imperial College London, London, SW7 2AZ UK; 2 Department of Natural Product Biosynthesis, Max Planck Institute for Chemical Ecology, 07745 Jena, Germany; 3 Université Paris-Saclay, INRAE, AgroParisTech, Micalis Institute, 78350 Jouy-en-Josas, France

**Keywords:** Crocetin, Zeaxanthin, β-Carotene, *Yarrowia lipolytica*, Secretory biosynthesis, Metabolic engineering, Synthetic biology, Microbial food

## Abstract

**Background:**

Crocetin is a multifunctional apocarotenoid natural product derived from saffron, holding significant promises for protection against various diseases and other nutritional applications. Historically, crocetin has been extracted from saffron stigmas, but this method is hindered by the limited availability of high-quality raw materials and complex extraction processes. To overcome these challenges, metabolic engineering and synthetic biology can be applied to the sustainable production of crocetin.

**Results:**

We constructed a *Yarrowia lipolytica* strain using hybrid promoters and copy number adjustment, which was able to produce 2.66 g/L of β-carotene, the precursor of crocetin. Next, the crocetin biosynthetic pathway was introduced, and we observed both the production and secretion of crocetin. Subsequently, the metabolite profiles under varied temperatures were studied and we found that low temperature was favorable for crocetin biosynthesis in *Y. lipolytica*. Therefore, a two-step temperature-shift fermentation strategy was adopted to optimize yeast growth and biosynthetic enzyme activity, bringing a 2.3-fold increase in crocetin titer. Lastly, fermentation media was fine-tuned for an optimal crocetin output of 30.17 mg/L, bringing a 51% higher titer compared with the previous highest report in shake flasks. Concomitantly, we also generated *Y. lipolytica* strains capable of achieving substantial zeaxanthin production, yielding 1575.09 mg/L, doubling the previous highest reported titer.

**Conclusions:**

Through metabolic engineering and fermentation optimization, we demonstrated the first de novo biosynthesis of crocetin in the industrial yeast *Yarrowia lipolytica.* In addition, we achieved a higher crocetin titer in flasks than all our known reports. This work not only represents a high production of crocetin, but also entails a significant simultaneous zeaxanthin production, setting the stage for sustainable and cost-effective production of these valuable compounds.

**Supplementary Information:**

The online version contains supplementary material available at 10.1186/s13068-024-02598-y.

## Background

Saffron, renowned as “the red gold” [1], is derived from the dried red stigmas of *Crocus sativus *L., a member of the *Iridaceae* family [2]. Its multifunctional natural products have been valued for over 4000 years [3] as a traditional remedy for more than 90 medical indications [4]. Among the various saffron-derived natural products, crocetin stands out as a widely investigated and reported main component [5]. This compound has demonstrated multiple bioactivities, including cardiomyocyte protection [6], hepatoprotection [7], neuroprotection [8], retinoprotection [9], anti-depression [10], anti-cancer [11], anti-diabetics [12] and anti-inflammatory actions [13]. Currently, the majority of crocetin is still extracted and purified from saffron stigmas [14, 15]. However, the limited availability of high-quality plant materials for extraction [16] and complex extraction processes from natural sources [17] present significant obstacles to large-scale production and application of crocetin and its derivatives. To address this challenge, metabolic engineering and synthetic biology emerge as promising strategies for achieving more sustainable and cost-effective microbial production of crocetin [18].

**Fig 1:**
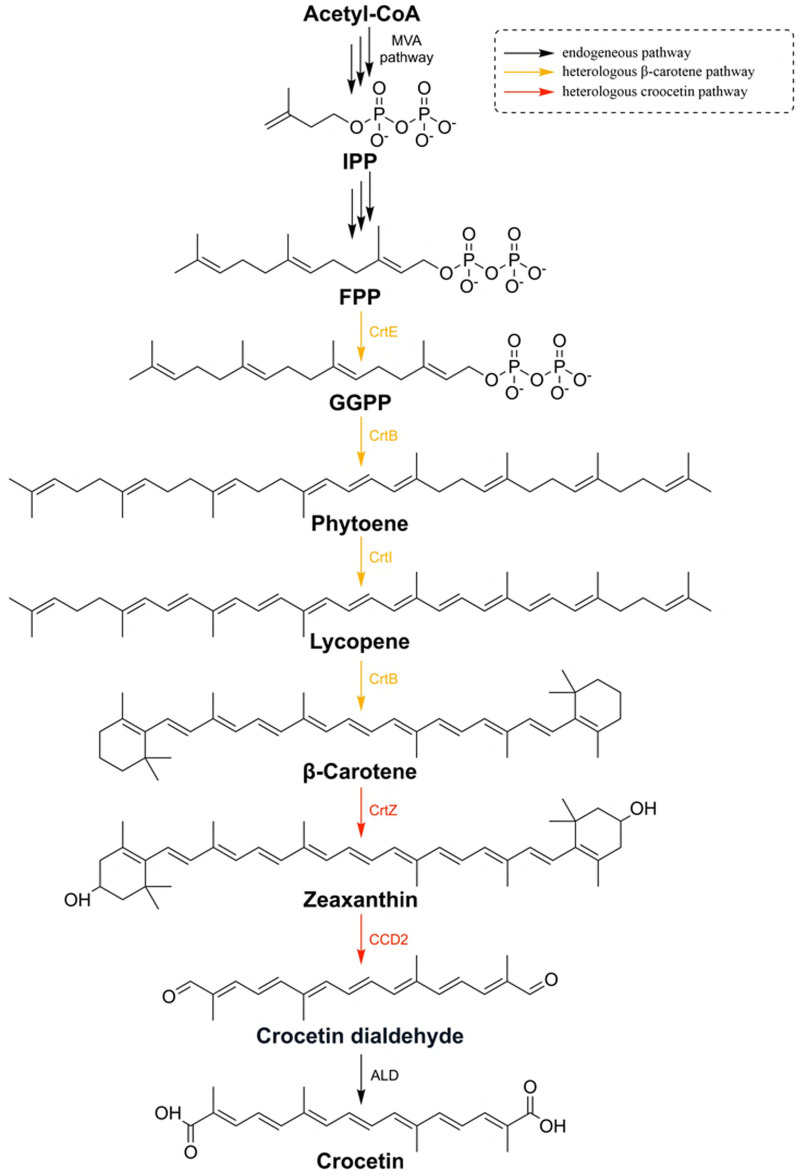
The crocetin biosynthetic pathway constructed in yeast. Acetyl-CoA: acetyl coenzyme A; IPP: isopentenyl diphosphate; FPP: farnesyl pyrophosphate; GGPP: geranylgeranyl pyrophosphate; GGS1: geranylgeranyl diphosphatase synthase: CarRP: bifunctional phytoene synthase/lycopene cyclase; CarB: phytoene dehydrogenase

The biosynthesis of crocetin typically begins with β-carotene [14, 19, 20] or zeaxanthin [15]. β-Carotene hydroxylase CrtZ [14], carotenoid-cleaving dioxygenase CCD2 [21], and aldehyde dehydrogenase ALD [14] sequentially convert them into crocetin (Fig. 1). The possibility of heterologous crocetin biosynthesis was first demonstrated in transgenic *Chlorella vulgaris* [22]. Quantitative microbial productions were also reported in *Saccharomyces cerevisiae* [14] and *Escherichia coli* [15]. Different strategies have been applied to improve the crocetin titer, as depicted in Additional file 1: Figure S1. To be more specific, different sources of CrtZ and CCD were screened (Additional file 1: Fig. S1a), and their copy numbers were adjusted to improve the crocetin production in *S. cerevisiae* which resulted in a 1.9-fold titer (Additional file 1: Fig. S1b) [14]. Engineering key enzyme, CCD2, by fusion with CrtZ or mutation (S323A) also resulted in the increase of crocetin titer by 49% [20] or 12.83-fold greater catalytic efficiency [19] in *S. cerevisiae* (Additional file 1: Fig. S1c and S1d). Considering the low bioactivity of CCD2 at 30 °C, a temperature-responsive promoter was applied in *E. coli* resulting in 139.67 µg/g DCW of crocetin (Additional file 1: Fig. S1e) [23]. Besides, improving the acetyl-CoA pool by deleting the genes involved in the glyoxylate cycle was also demonstrated as a helpful strategy to facilitate crocetin biosynthesis (Additional file 1: Fig. S1f) [20]. Despite the application of various metabolic engineering strategies and extensive research on the model microorganisms *E. coli* and *S. cerevisiae*, crocetin production still remains highly restricted (Table 1). The limited progress in biosynthesis could be attributed to the lack of suitable microbial chassis [24] and choosing the appropriate starting strain which is a critical aspect of achieving successful industrial production [25].

**Table 1:** Overview of microbial biosynthesis of crocetin

Microbial chassis	Crocetin titer	References
*Y. lipolytica*	30.17 mg/L in flask	This work
*E. coli*	4.42 mg/L in flask	[15]
*E. coli*	477.15 µg/L in flask 34.77 mg/L in 5-L bioreactor	[49]
*S. cerevisiae*	1.22 mg/L in flask 6.28 mg/L in 5-L bioreactor	[14]
*S. cerevisiae*	1.95 mg/L in flask 12.43 mg/L in 5-L bioreactor	[20]
*S. cerevisiae*	139.67 µg/g DCW in flask	[23]
*S. cerevisiae*	20 mg/L in flask 107 mg/L in 5-L bioreactor	[19]

*Yarrowia lipolytica* has gained interest as an oleaginous yeast in both academia and industry due to its outstanding ability to manufacture high-yield products [25]. The lipophilic nature of β-carotene derivatives promotes their storage in yeast lipid bodies [26], making *Y. lipolytica* a perfect candidate for their biosynthesis. For example, *Y. lipolytica* has been engineered to achieve high-yield production of various carotenoids such as β-carotene [27], zeaxanthin [28], astaxanthin [29], β-ionone [30] and retinol [31]. This exceptional capability stems from its proficient metabolic flux towards acetyl-CoA, which secures the abundance of the precursors isopentenyl diphosphate (IPP)/dimethylallyl diphosphate (DMAPP) [24]. Given that crocetin also belongs to the apocarotenoid family [32], *Y. lipolytica* has the potential to supply ample IPP/DMAPP precursors for its biosynthesis. Hence, in this study, we employed *Y. lipolytica* for the first time to explore its capacity for crocetin production. Moreover, *Y. lipolytica* is recognized as a generally recognized as safe (GRAS) strain [33], making it an ideal candidate for producing crocetin as a safe and viable option for food and medical applications.

In this study, a β-carotene producer was constructed to provide a large pool of precursors for crocetin through the use of hybrid promoters and copy number adjustment. Subsequently, we introduced the crocetin cassette to the best β-carotene producer. Interestingly, we observed crocetin secretion, which has not been reported so far. A two-step temperature-shift fermentation strategy further enhanced the crocetin titer. Additionally, fermentation optimization was also applied to increase crocetin production.

## Materials and methods

### Strains and media

*Escherichia coli* Top10 was used for cloning and plasmid propagation. *E. coli* cells were cultivated in 3 mL LB medium (VWR) at 37 °C in the 250-rpm constant-shaking incubator. When needed, 100 µg/mL chloramphenicol (Chl), 150 µg/mL spectinomycin (Spec), or 100 µg/mL ampicillin (Amp) were added to LB agar (EMD Millipore) for selection.

*Yarrowia lipolytica* YB392 was used as the parental yeast strain. *Yarrowia lipolytica* cells were cultivated in 3 mL YPD media composed of 20 g/L glucose (VWR), 20 g/L peptone from casein (EMD Millipore), and 10 g/L yeast extract (Sigma-Aldrich) in 24-well deep well plates (EnzyScreen) at 30 °C or 20 °C in the 250-rpm constant-shaking incubator. The initial starting OD_600_ for yeast cultivation was 0.5. When needed, YNBD agar composed of 1.7 g/L YNB without amino acid and ammonium sulfate (Sigma-Aldrich), 5 g/L NH_4_Cl (VWR), 20 g/L glucose (VWR), 3.87 g/L NaH_2_PO_4_ (VWR), 3.76 g/L Na_2_HPO_4_(VWR) and 20 g/L agar (EMD Millipore) were used for yeast selection after transformation, and 100 mg/L leucine (Leu, Sigma-Aldrich) or 160 mg/L tryptophan (Trp, Sigma-Aldrich) were added based on auxotrophic types.

### Construction of plasmids and strains

All genes *GGS1, CarRP, CarB*, *CrtZ*, *CCD2,* and *ALD* (Additional file 1: Table S1) were synthesized by Twist Bioscience after codon optimization for *Y. lipolytica* and BsaI/BsmBI/NotI restriction enzyme cutting sites were avoided. All related plasmids were constructed using Golden Gate (GG) based *Yarrowia* Toolkit (YaliCraft) [34], which was developed from YTK Toolkit [35] and adapted for *Y. lipolytica* in 2017 [36]. GG assemblies were performed in a 10 µL reaction system: 50 fmol/µL DNA parts, 1 µL T4 ligase buffer (NEB), 0.5 µL T7 ligase (NEB), 0.3 µL BsmBI/BsaI (NEB) and ddH_2_O up to 10 µL and the following thermal profile was applied for GG assembly: 90 cycles of 37 °C for 2 min and 16 °C for 5 min, followed by 60 °C for 10 min and 80 °C for 10 min. All the sequences to be used as integration blocks after GG assembly were sequenced by Source Bioscience (Cambridge, UK) or verified by restriction enzyme digestion.

GoTaq Green Master Mix (Promega) and Phire Plant Direct PCR Kit were used for cloning PCR for *E. coli* and *Y. lipolytica*, respectively. All polymerase chain reactions were performed according to the manufacturer's manual. When needed, PCR products were purified by Monarch PCR & DNA Cleanup Kit or Gel Extraction Kit (NEB). Primers were synthesized by IDT. All primers used in this study are listed in Additional file 1: Table S2.

Chemical transformation of plasmid DNA into *E. coli* competent cells were performed using the heat shock method. *E. coli* cells after transformation were selected on LB-Chl, LB-Spec, or LB-Amp agar plates. Chemical transformation of plasmid DNA into *Y. lipolytica* cells was performed in 100 µL volume: 1.5 pmol NotI (NEB) digested plasmids, 85 µL 50% PEG 4000 (Sigma-Aldrich), 5 µL 2M LiAc (VWR), 5 µL 2M DTT (Thermo Scientific), 5 µL salmon sperm carries DNA (Invitrogen) and a loop of cells. The mixed solution was incubated at 30 °C for 30 min and heat shocked at 42 °C for 10 min. Then, transformants were selected on YNBD-Leu or YNBD-Trp agar plates. All the plasmids and yeast strains used in this study are listed in Additional file 1: Table S3.

Plasmid pUB4-Cre [37] was transformed into targeted *Y. lipolytica* cells to rescue LEU2 and TRP4 markers. Transformants were selected on YPD agar plates with 200 mg/L hygromycin B (Hygro, Invitrogen). Colonies were grown on YPD-Hygro agar plates for 2-3 days and then transferred to YPD agar plates. Correct colonies with successful marker deletion were confirmed by sub-cultivation on YNBD-Leu, YNBD-Trp, and YNBD-Leu-Trp agar plates. Verified colonies were then cultivated in YPD media, ensuring complete loss of pUB4-Cre before further metabolic engineering.

The strain construction tree in this research can be found in Additional file 1: Figure. S2.

### Extraction and quantitative analysis of carotenoids

Cell pellets from 500 µL culture broth were collected in 2 mL screwcap microtubes (SARSTEDT) by centrifugation at 8000 rpm for 3min after fermentation for intracellular β-carotene or zeaxanthin extraction. 200 µL acid-washed glass beads (425–600 µm, Sigma-Aldrich) and 500 µL acetone (VWR) were added to the microtube, and microtubes were put into Precellys Evolution homogenizer (Bertin Technologies) for cell crushing. The homogenizer was set 3 cycles at 8000 rpm for 1 min with intervals of 15 s. The extract was collected by centrifugation at 12000 rpm for 1 min, and the extraction procedure was repeated until the pellet and the supernatant were colorless. All the extract was mixed and analyzed by high-performance liquid chromatography (HPLC, Vanquish Core, Thermo Scientific) equipped with a Carotenoid C30 column (150 mm × 4.6 mm, 3 µm, TMC) and a UV detector. The signals were detected at 455 nm, and the column temperature was set at 25 °C. Solvent system: acetonitrile (ACN), methanol (MeOH), and methyl *tert*-butyl ether (MTBE). Isocratic 3% ACN and 97% MeOH (0–3 min); gradient 3–10% of ACN, 97–85% MeOH and 0–5% MTBE (3–5 min); gradient 10–15% of ACN, 85–75% MeOH and 5–10% MTBE (5–15 min); gradient 15–40% of ACN, 75–45% MeOH and 10–20% MTBE (15–30 min); isocratic 40–3% of ACN, 40–97% MeOH and 20–0% MTBE (30–40 min). The flow rate was set at 1.0 mL/min. β-carotene standard was purchased from Sigma-Aldrich (standard curve in Additional file 1: Fig. S3a), and the zeaxanthin standard was purchased from Yuanye Biotech (standard curve in Additional file 1: Fig. S3b).

Cell pellets from 2 mL culture broth were collected in 2-mL screwcap microtubes (SARSTEDT) by centrifugation at 8000 rpm for 3min after fermentation for intracellular crocetin extraction. 200 µL acid-washed glass beads (425–600 µm, Sigma-Aldrich) and 500 µL acetone (VWR) were added to the microtube, and microtubes were put into Precellys Evolution homogenizer (Bertin Technologies) for cell crushing. The homogenizer was set 8 cycles at 8000 rpm for 1 min with intervals of 15 s. The extract was collected by centrifugation at 12000 rpm for 1 min, and the extraction procedure was repeated until the pellet and the supernatant were colorless. All the extract was mixed, concentrated in concentrator plus (Eppendorf) at 60 °C for vacuum concentration, and re-dissolved in 200 µL 50% acetone. C18 column (150 × 4.6 mm, 120 Å, 5 µm, Acclaim) was used to characterize crocetin by HPLC, and crocetin was detected at 430 nm. The mobile phase consisted of 70% MeOH-containing 2% formic acid with a 1.0 mL/min flow rate at 40 °C. Crocetin standard was purchased from Sigma-Aldrich (standard curve in Additional file 1: Fig. S3c).

For carotenoids secreted to the medium, 500 µL culture broth was collected by centrifugation at 8000 rpm for 3 min, concentrated, and analyzed by HPLC following the methods above.

### Flask fermentation and optimizations

50-mL Erlenmeyer flasks (VWR) were adopted for carotenoid flask fermentation. Initial OD_600_ was also set at 0.5, and *Y. lipolytica* cells after pre-culture were inoculated into 10 mL media and cultured with a rotation speed of 250 rpm. Media optimization was based on YPD (Additional file 1: Table S4). The fermentation temperature for crocetin was set at 30 or 20 °C.

### Growing curve and dry biomass

The culture broth was collected and diluted with dd H_2_O 10–200 times, and fresh YPD media were used as a blank with the same dilution. 500 µL culture broth was collected and washed with dd H_2_O 3 times to discard media compositions and then put in a 60 °C drying oven in pre-measured 1.5-mL tubes. After 48 h, tubes with dry biomass were measured, and dry cell weight (DCW) was calculated.

### Wave scanning of supernatant after fermentation

Spectrophotometer (WPA Biowave II, Biochrom) was used to detect the absorbance of culture broth after fermentation from 400 to 500 nm. Medium after fermentation was collected by centrifugation at 8000 rpm for 3 min and diluted 4 times before detection. Absorbance was tested at different wavelengths, and wavelengths were changed with an interval of 5–10 nm. Fresh YPD medium was used as a blank.

### Microscopy and flow cytometry

Cells were collected after fermentation and tenfold diluted in PBS. Cells were stained using a final concentration of 0.01% Calcofluor White and 1 µg/mL propidium iodide for 5 minutes at room temperature. Cells were then transferred to microscopy slides for imaging. Images were acquired using a Nikon Eclipse Ti2 microscope fitted with a Prime BSI sCMOS camera. 21 Z-stacks were taken per image with a total depth of 7 µm using a 60x oil-immersion objective. Blue (Calcofluor White) and red (propidium iodide) fluorescence were detected at constant exposure times and light intensities. Images were prepared using ImageJ/FIJI.

For flow cytometry, cells were diluted hundred-fold and stained with 1 µg/mL Propidium Iodide for 5 min at room temperature. Fluorescence was detected using an Attune Flow Cytometer and cell death was monitored using the yellow 561 nm laser with a 620/15 bandpass filter. Data were gated and analyzed in R Studio.

### Statistical analysis

All data in this study were represented as mean ± standard deviation (SD) from 3 biological replicates. Dunnett’s and Šídák's multiple comparison tests were conducted using GraphPad Prism (Version 9.3.0). *ns*:*p* > 0.05, ^*^p ≤ 0.05, ^**^p ≤ 0.01, ^***^p ≤ 0.001, ^****^p ≤ 0.0001. *p* ≤ 0.05 was defined to be statistically significant in the statistical analysis.

## Results

### Constructing the optimal chassis strain for abundant β-carotene precursor pool

*Yarrowia lipolytica* has proven its efficacy in the production of various carotenoids, including β-carotene [27], zeaxanthin [28], astaxanthin [29], β-ionone [30] and retinol [31]. In previous studies, distinct strains of *Y. lipolytica*, such as ATCC MYA2613, Po1d, Po1f, Po1h, and CJ1025, derived from the common isolate *Y. lipolytica* W29 (ATCC 20460), have served as parental strains.

**Fig. 2:**
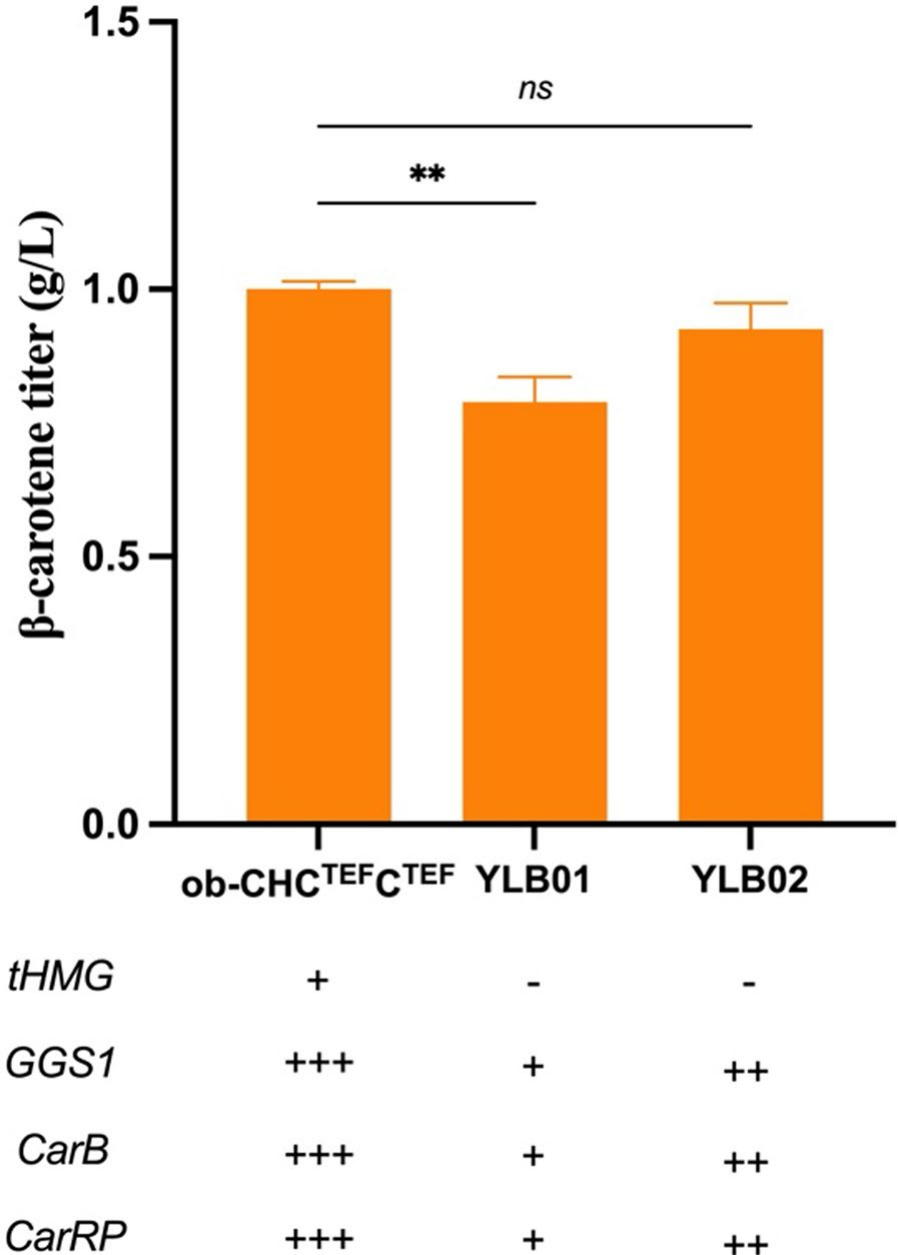
Comparison of β-carotene titer between different *Y. lipolytica* strains. All strains were cultured at 30 °C for 4 days

Previous research utilized the *Y. lipolytica* Po1d-derived mutant, JMY3501, specifically engineered to enhance lipid accumulation, as the parental strain for β-carotene biosynthesis [26]. This lipid-overexpressing strain (ob-CHC^TEF^C^TEF^) exhibited superior performance in terms of β-carotene titer, likely attributed to an augmentation of lipophilic structures within the cells for efficient carotenoid storage [26]. Among various *Y. lipolytica* strains, YB392 has been validated for its superior lipid biosynthesis compared to W29 [38], suggesting a potential enhancement in its ability to biosynthesize β-carotene, the precursor of crocetin. Consequently, we first tested the capacity of YB392 to produce β-carotene (Fig. 2). Here, *GGS1*, *CarB*, and *CarRP* were expressed under the control of TEF promoter to construct the β-carotene pathway in YB392, resulting in 0.79 g/L of β-carotene production in YLB01 after 4 days of cultivation (Fig. 2). Upon increasing the copy number of biosynthetic genes, the β-carotene titer increased to 0.93 g/L after the 4-day cultivation (YLB02). The β-carotene titer from YLB02 is comparable to the one from the previously engineered strain ob-CHC^TEF^C^TEF^ (1.00 g/L) (Fig. 2). Notably, despite achieving a similar β-carotene titer, YLB02 required less genetic modifications and gene dosage, which indicates a higher potential to produce more carotenoids (Fig. 2). Given these findings, we selected YB392 as the parental strain for crocetin biosynthesis.

### Increasing the expression level of genes to improve β-carotene production

In the majority of reported crocetin studies utilizing *S. cerevisiae* as the microbial chassis [14, 19, 20], crocetin biosynthesis was built upon a β-carotene-producing strain with a β-carotene titer of 220 mg/L. However, this β-carotene level may not be sufficient to provide abundant precursors for efficient downstream crocetin biosynthesis. While the above-described strain YLB02 produced higher amounts, further increasing β-carotene could lead to higher titers of crocetin.

**Fig. 3:**
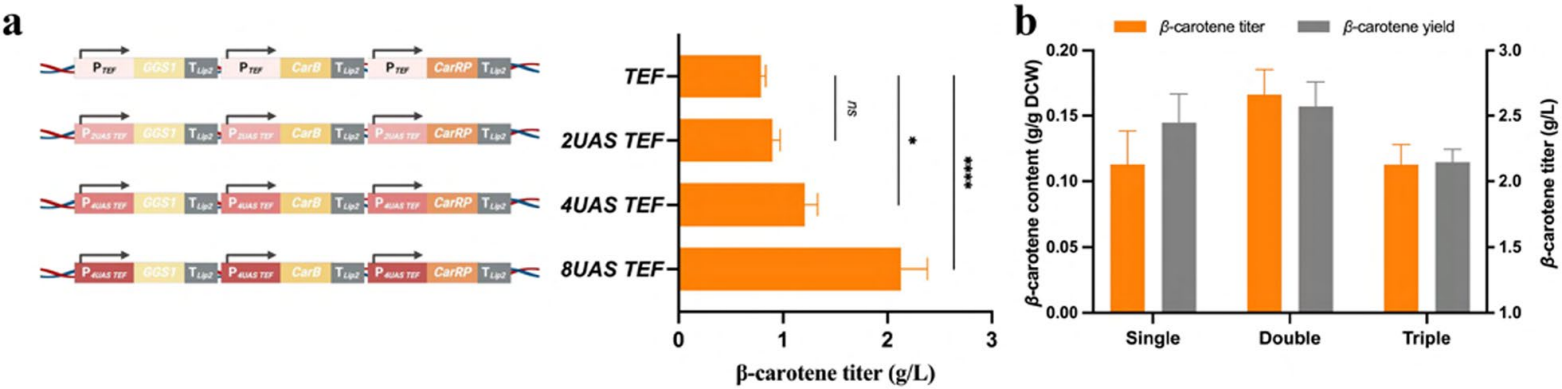
Enhancing β-carotene production by increasing gene expression level. **a** Influences of using hybrid promoter to enhance β-carotene titer; **b** influences of copy number on β-carotene titer and content from the producer under P_*8UAS TEF*_ control. All strains were cultured at 30 °C for 4 days. DCW, dry cell weight

Previously, the TEF1 promoter has been identified as the strongest promoter for β-carotene production in *Y. lipolytica* through promoter shuffling, where the strain carrying three genes (*GGS1-CarB-CarRP*) under TEF1 promoter demonstrated optimal β-carotene production [26]. Therefore, TEF1 was chosen as the initial promoter for constructing β-carotene producers in this study. To further enhance the promoter strength, hybrid promoters harboring different numbers of upstream activation sequences (UAS) of XPR2 promoter and core TEF were employed [39] (Fig. 3a). As the number of UAS increased, the β-carotene titer was improved correspondingly (Fig. 3a). The highest β-carotene titer, 2.13 g/L, was obtained from the expression under 8UAS TEF promoter after 4 days of cultivation (Fig. 3a).

Considering that gene copy number is another critical factor affecting carotenoid titer [31, 40–42], it was further optimized by integrating multiple copies of the expression cassette into the genome of the previous best β-carotene producer. The highest titer of 2.66 g/L was achieved through double transformation (YLB11), and no significant improvement was observed with a triple one (Fig. 3b). Based on these results, the strain YLB11 was selected as a parent strain for crocetin biosynthesis.

### Engineered Y. lipolytica can produce and secrete crocetin

**Fig. 4:**
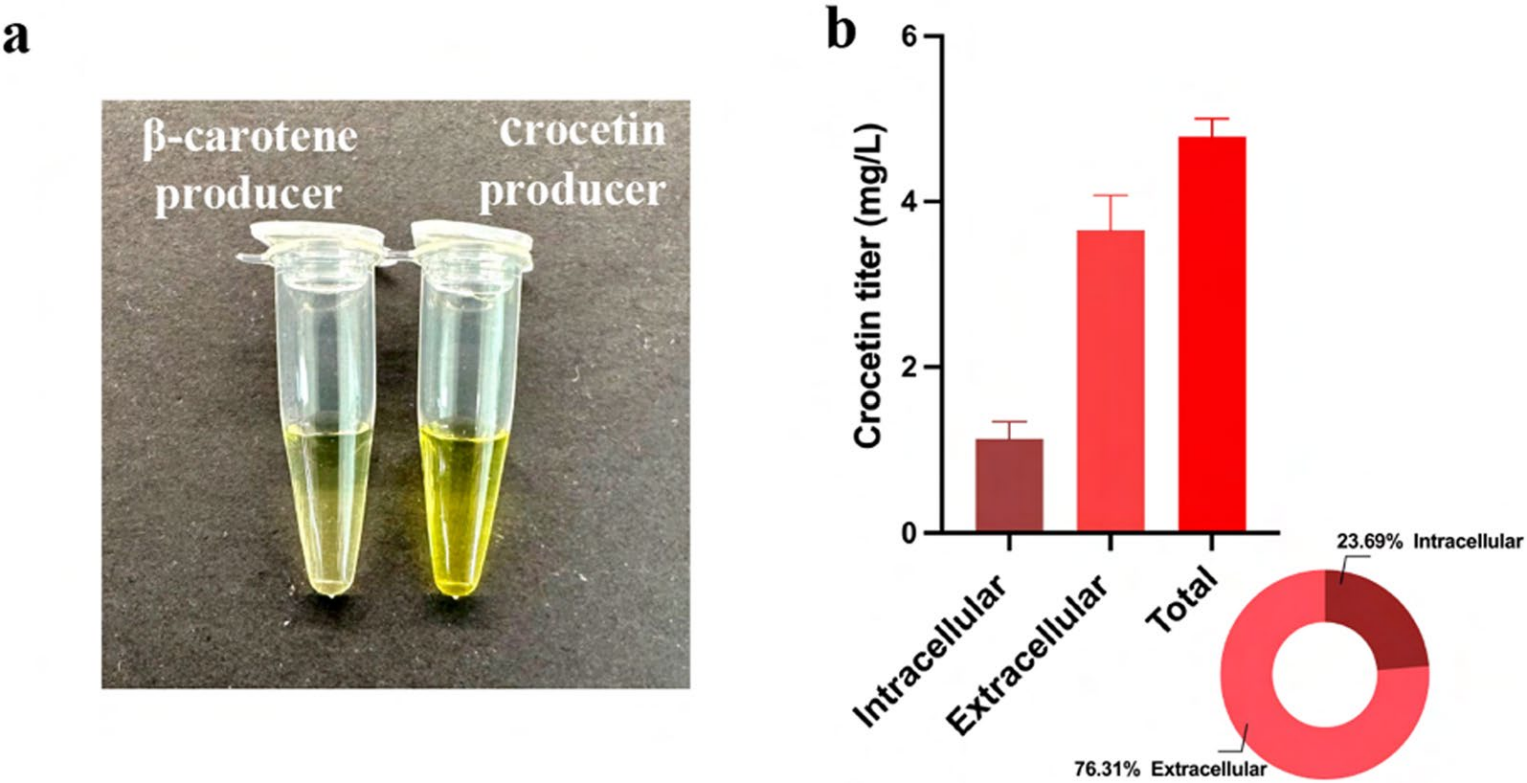
Secretory production of crocetin in *Y. lipolytica*. **a** Supernatant of β-carotene and crocetin producer after 7 days fermentation; **b** distribution of crocetin in *Y. lipolytica* after fermentation at 30 °C for 7 days

Two enzymes, β-carotene hydroxylase (CrtZ) and carotenoid-cleaving dioxygenase (CCD2), are necessary for crocetin biosynthesis from β-carotene (Fig. 1). CrtZ from *Pantoea stewartia* and CCD2 from *C. sativus* were expressed under 8UAS TEF promoter in the β-carotene-producing strain YLB11. Surprisingly, we observed a deeper yellow color of cultivation broth compared to the supernatant of the β-carotene producer (Fig. 4a). We detected supernatants’ ultraviolet–visible (UV–vis) absorption spectrum between 400 and 500 nm and two main peaks with absorption wavelengths at 420 and 445 nm were observed (Additional file 1: Fig. S4). Then, the pigmented product in the crocetin producer’s supernatant was verified as crocetin by high-performance liquid chromatography (HPLC) (Additional file 1: Fig. S5a). The majority of crocetin (3.65 mg/L) was found to be secreted to the culture medium by *Y. lipolytica*, and 23.69% (1.13 mg/L) of total crocetin remained in the cell (Fig. 4b), which was a phenomenon not observed in previous reports [14, 15, 19, 20, 22].

In this study, a significant amount of crocetin was detected in the media (Fig. 4b). To rule out the possibility that extracellular crocetin resulted from potential cell lysis, the distribution of both β-carotene and zeaxanthin was also analyzed, which would also be present extracellularly if cell lysis occurred. We conducted separate detections of these compounds in both extracellular and intracellular environments (Additional file 1: Fig. S5). The results revealed that β-carotene and zeaxanthin were exclusively intracellularly accumulated, with no detectable levels in the extracellular media (Additional file 1: Fig. S5b). This finding suggests that the presence of extracellular crocetin is not a result of cell lysis but rather a result of secretion.

### Carotenoid profiles of crocetin producers under different temperatures

**Fig. 5:**
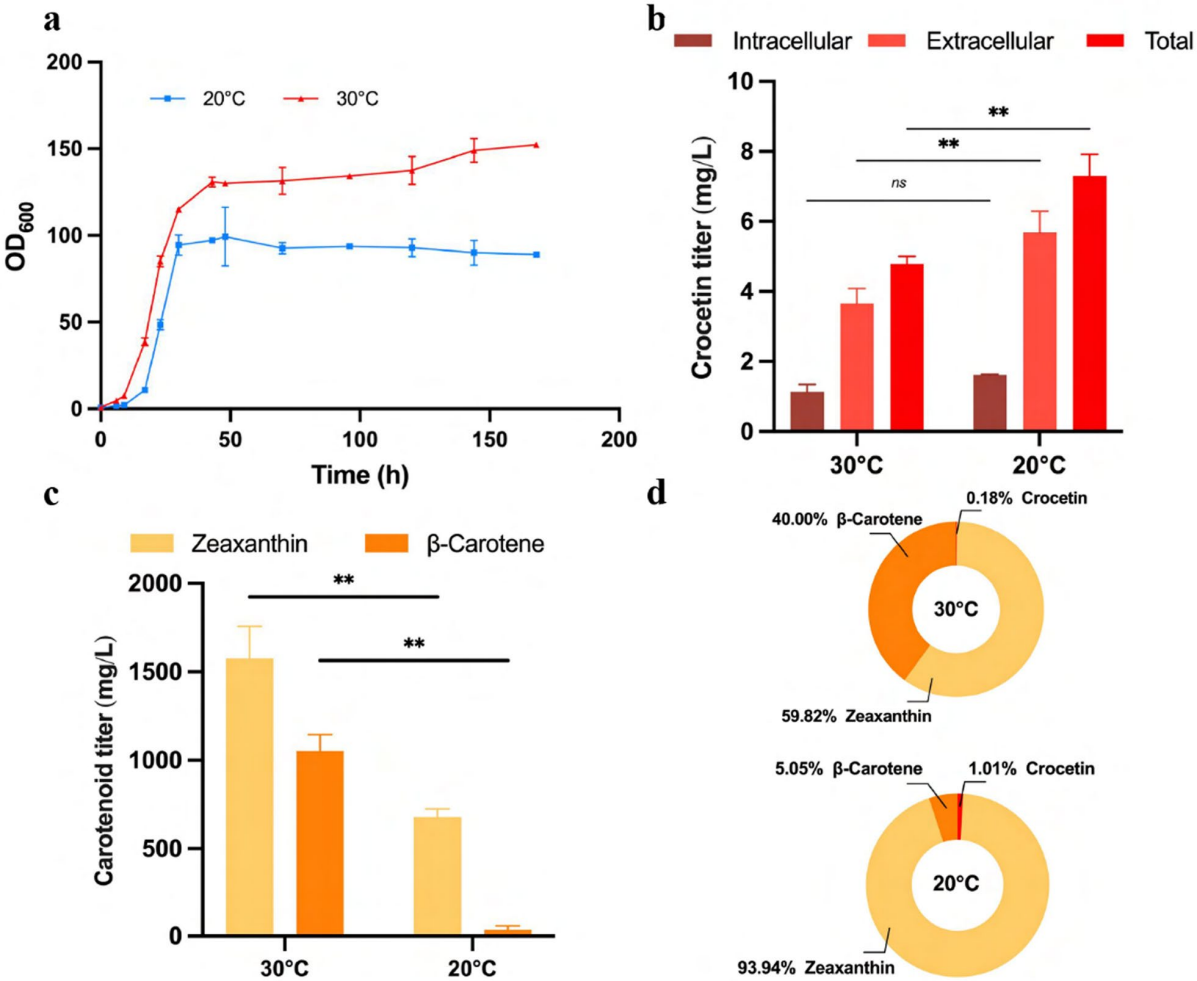
The effect of cultivation temperature on the production of crocetin, zeaxanthin, and β-carotene. **a** Growth curve of crocetin-producing strain under different temperatures; **b** crocetin titer under different temperatures; **c** zeaxanthin, and β-carotene titer under different temperatures; **d** metabolic profiles under different temperatures (molar ratio)

Distinct from the intracellular production of crocetin in *S. cerevisiae* described so far, *Y. lipolytica* generated crocetin as both intracellular and extracellular products. The rate-limiting step in crocetin biosynthesis is the CCD2-catalyzed reaction, as previously established [14, 19]. Remarkably, CCD2 exhibited minimal intracellular catalytical activity at 30 °C, but optimal crocetin production was observed at 20 °C in *S. cerevisiae* [14]. To verify if a lower temperature is favorable for crocetin biosynthesis, we cultivated the strain at 20 °C. The cultivation at 20 °C led to a notable reduction in biomass, dropping OD_600_ from approximately 150 to below 90 (Fig. 5a). After a 7-day fermentation, extracellular crocetin concentration from the cultivation at 20 °C was 5.68 mg/L which is 1.56 times of the one at 30 °C (Fig. 5b). Intracellular crocetin concentration also increased slightly from 1.13 mg/L to 1.62 mg/L (Fig. 5b). In total, crocetin titer increased from 4.78 mg/L to 7. 30 mg/L after decreasing the temperature from 30 °C to 20 °C (Fig. 5b). The elevated levels of both extracellular and total (Fig. 5b) titers at low temperature are aligned with the previous study showing higher production at 20 °C in *S. cerevisiae* [14].

The production of key precursors of crocetin, zeaxanthin, and β-carotene was also different depending on culture temperatures (Fig. 5c and d). Zeaxanthin titers at 30 °C and 20 °C were 1575.09 and 678.93 mg/L, respectively, while β-carotene titers were 1053.23 and 36.47 mg/L (Fig. 5c). At the same time, the crocetin molar ratio was increased from 0.18 to 1.01% after lowering the temperature from 30 to 20 °C (Fig. 5d). The distinct colors of the culture broth also visually indicated their disparate metabolite levels (Additional file 1: Fig. S6).

However, regardless of the temperatures, zeaxanthin consistently remained the dominant metabolite among β-carotene, zeaxanthin, and crocetin (Fig. 5d), highlighting the conversion of zeaxanthin into crocetin as a bottleneck in the crocetin biosynthesis. Thus, within *Y. lipolytica*, the CCD2-catalyzed reaction continued to serve as the limiting factor, aligning with earlier findings in *S. cerevisiae* [14, 19].

### Two-step temperature-shift fermentation for an enhanced crocetin titer

Previously, in both *S. cerevisiae* and *E. coli*, crocetin production was undertaken at 20 °C [14, 15, 19, 20], despite its unsuitability for optimal growing conditions. Inspired by Liu’s research [23], a novel two-step temperature-shift fermentation strategy was conducted aiming at balancing yeast growth and crocetin accumulation. Similar temperature-shift strategies have already been successfully employed in other organisms to enhance product biosynthesis, such as the enhancement of l-lactic acid production in *Lactiplantibacillus plantarum* [43] and lutein biosynthesis in *S. cerevisiae* [44].

**Fig. 6:**
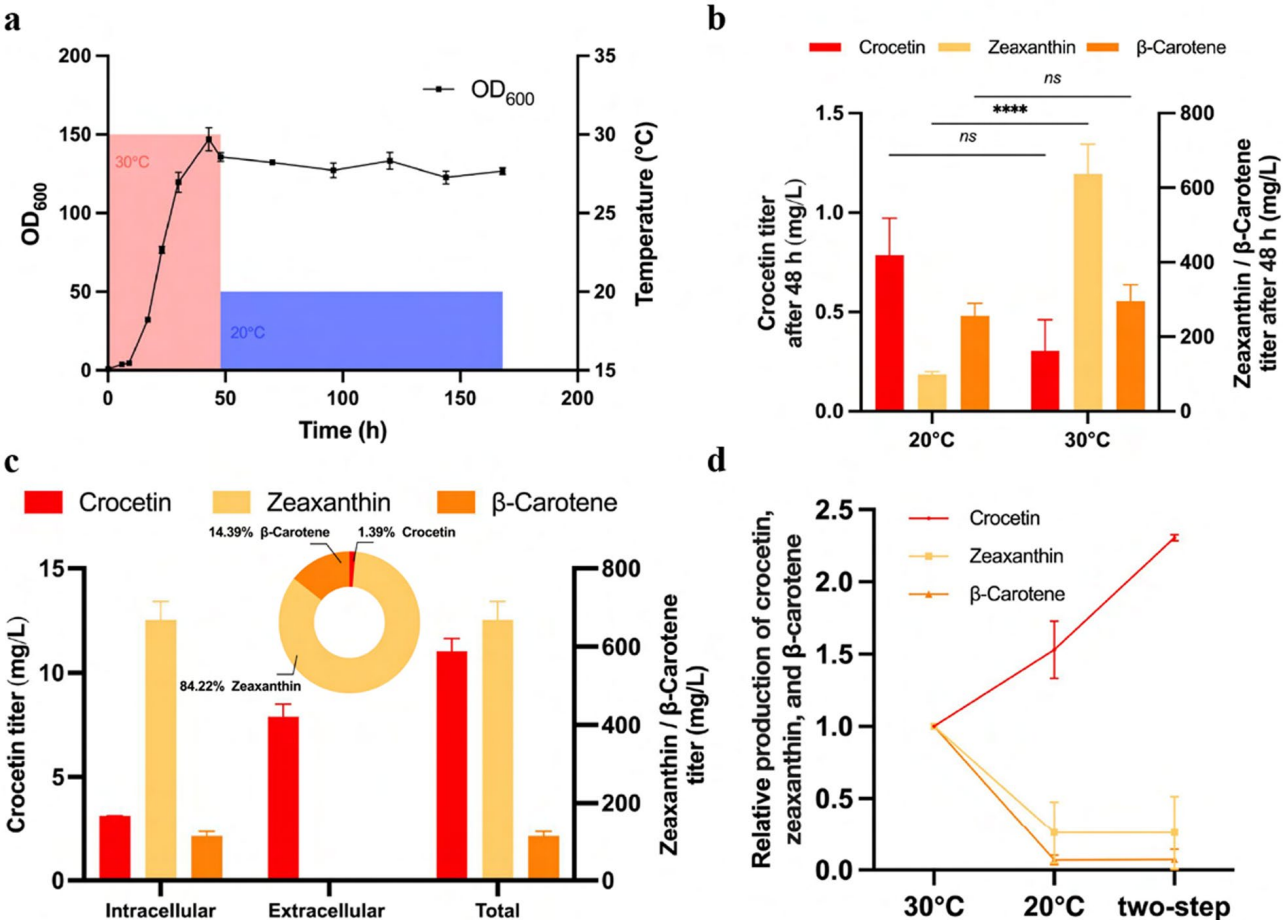
Crocetin producers cultivated with the two-step temperature-shift strategy. **a** Growth curve of crocetin producer during cultivation with a temperature shift; **b** total titer of crocetin, zeaxanthin, and β-carotene cultivated after 48 h of cultivation at 20 and 30 °C; **c** crocetin, zeaxanthin, and β-carotene titers with two-step temperature-change strategy after 7 days of cultivation; **d** relative carotenoid profile changes compared with 30 °C fermentation

The yeast cultivation was initiated at 30 °C for the initial 2 days, facilitating a rapid progression to the stationary phase and achieving an OD_600_ of approximately 126 (Fig. 6a), far exceeding the final biomass achieved at 20 °C (Fig. 5a). To check whether it was the suitable time point for the temperature shift, biomass after 2-day cultivation at 20 and 30 °C was collected (Fig. 6b). Although a slightly higher mean value of crocetin titer was observed in the 20 °C group, the 30 °C one resulted in a significant higher zeaxanthin titer (Fig. 6b), indicating substantial potential for subsequent crocetin production, further supporting our strategy to adopt two-step fermentation instead of the constant 20 °C one. Ultimately, the two-step fermentation process attained a crocetin titer of 3.13 mg/L intracellularly and 7.90 mg/L extracellularly (Fig. 6c). When compared with fermentation at 30 °C after 7 days, zeaxanthin and β-carotene titers decreased by 2.34- and 9.21-fold, respectively, while crocetin increased by 2.30-fold (Fig. 6d), underscoring the efficacy of two-step temperature-change fermentation strategy for crocetin production.

Crocetin producer’s viability was also monitored by morphological observation and flow cytometry to verify if the extracellular crocetin came from the cell lysis and compare the two different fermentation strategies (Additional file 1: Fig. S7). In the two-step temperature-change fermentation, only 2.12% cells were found to be dead (Additional file 1: Fig. S7a, b). In contrast, 24.91% of cells died after fermentation at 30 °C after 7 days (Additional file 1: Fig. S7c, d), indicating better cell viability using the two-step strategy. Furthermore, the staining experiments and cell viability statistics (Additional file 1: Fig. S7) strongly suggested that *Y. lipolytica* has the potential to secrete crocetin, and it is less likely that the extracellular crocetin is solely a result of cell lysis.

After optimizing the fermentation strategy, an exogenous ALD sourced from *Synechocystis* sp. PCC6803 (*Syn*_ALD), which has gained widespread usage for crocetin production [14, 19, 45], was also tested in our research. *Syn_ALD* was codon optimized for *Y. lipolytica* and integrated under the regulation of the 8UAS TEF promoter (Additional file 1: Fig. S8a). This introduced ALD did not show any positive effect on crocetin production (Additional file 1: Fig. S8b,c)

### Medium optimization to increase crocetin production

**Fig. 7:**
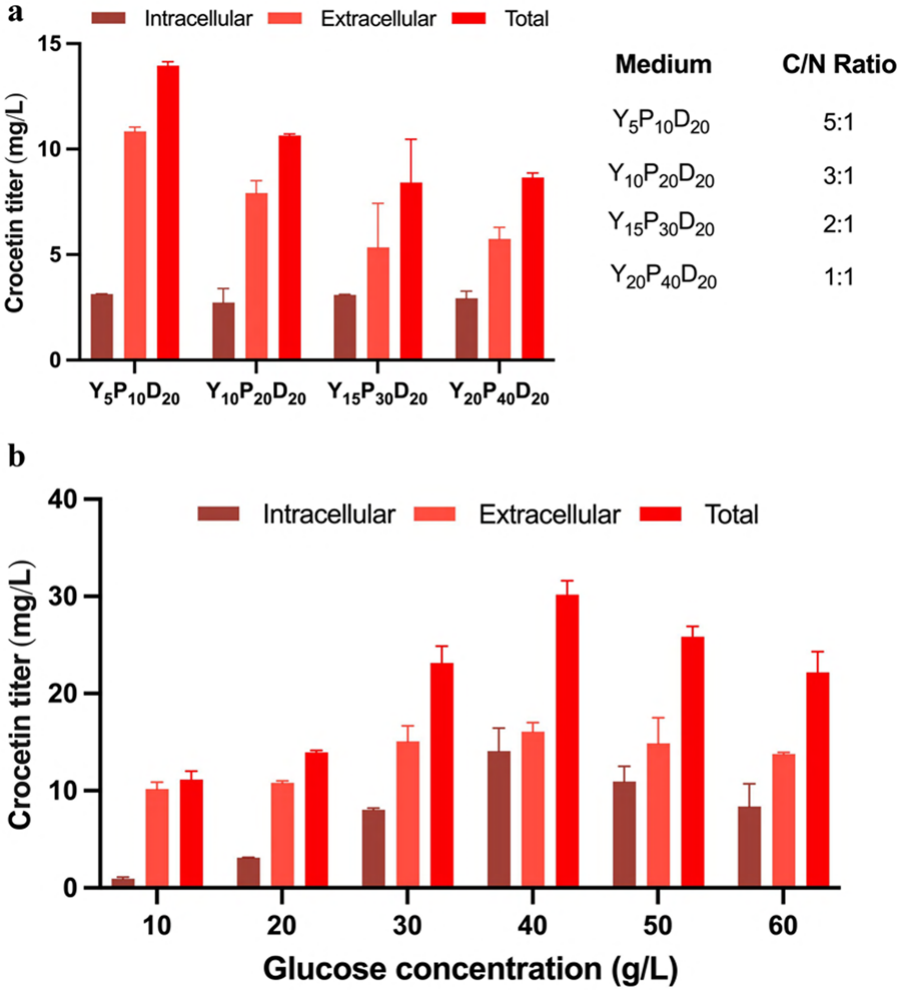
Medium optimization for crocetin titer in *Y. lipolytica*. **a** Effects of C/N ratio on crocetin titer; **b** effects of glucose concentration on crocetin titer. YLC01 was cultivated using the two-step fermentation method (2 days at 30 °C and then 5 days at 20 °C) when optimizing the C/N ratio and glucose concentration

Next, we decided to optimize the medium composition for the crocetin producer strain (YLC01) based on the two-step temperature-change strategy to increase crocetin production further. Initially, we explored the effects of the carbon/nitrogen (C/N) ratio on crocetin production (Fig. 7a). Consistent with existing literature, which suggests that a lower C/N ratio can facilitate carotenoid synthesis [27], we explored various low C/N ratios from 1:1 to 5:1 (Additional file 1: Table S4). This investigation led to the identification of an optimal C/N ratio of 5:1, yielding the highest crocetin concentration of 13.96 mg/L (Fig. 7a).

Based on this result, we fixed the C/N ratio and optimized glucose concentration (Additional file 1: Table. S4). The highest crocetin titer of 30.17 mg/L was achieved by using 40 g/L glucose as the carbon source (Fig. 7b). Notably, further increasing the glucose concentration to 50 or 60 g/L did not enhance, but rather reduced, the crocetin titer (Fig. 7b).

## Discussion

Considering the notable increase of β-carotene in our research, copy number adjustment might also be useful for improving crocetin production in future studies. Additionally, we observed the secretion of crocetin in *Y. lipolytica*, a phenomenon not previously reported in any microorganism. This could be attributed to *Y. lipolytica*’s inherent secreting capabilities, as evidenced by its secretory production of other terpenoids such as retinol [31], abscisic acid and gibberellic acid [46]. This insight indicates the potential for further increasing crocetin production through transporter engineering, a technique already applied in *S. cerevisiae* for β-carotene and retinal production.

To address the bottleneck catalyzed by CCD2, we applied a temperature-shift strategy in this research, which effectively boosted the crocetin titer by 2.30 times (Fig. 6). Future experiments combining temperature shifts with fed-batch fermentation in yeast could further enhance crocetin production. What’s more, improvement could also come from protein engineering of CCD2 to increase its catalytical activity and thermal stability [19]. Considering the recent identification of GjCCD4a from *Gardenia jasminoides*, which can catalyze crocetin dialdehyde from various substrates [47], broadening the enzyme screening for efficient crocetin biosynthesis might be helpful to address this rate-limiting step. Also, there is a contrast between the chemical properties of crocetin precursors (β-carotene and zeaxanthin) and crocetin itself: the precursors are lipophilic and tend to be accumulated in lipid bodies [26], whereas crocetin is more hydrophilic and can potentially be secreted into the medium. The precursors stored in lipid bodies may be less accessible to enzymes in the subsequent reaction converting zeaxanthin to crocetin. Targeting CCD2 to the lipid bodies might help to improve access to these precursors and potentially enhance conversion rates.

It was shown that our crocetin producers could accumulate a high amount of intracellular zeaxanthin of 1575.09 mg/L (Fig. 5c), which was even higher than the current best zeaxanthin reports in *E. coli* (722.46 mg/L achieved by 5 L fed-batch) [48] and *Y. lipolytica* po1h (775.3 mg/L in YPD shake flasks) [28]. This indicated the engineered strains constructed in this study also have a great potential for zeaxanthin biosynthesis.

## Conclusion

In this study, we initially engineered a high-yield β-carotene-producing strain, achieving a concentration of 2.66 g/L through hybrid promoter utilization and copy number adjustments, thereby supplying ample precursors for crocetin. Subsequently, we introduced the crocetin biosynthetic pathway into *Y. lipolytica*, realizing the de novo crocetin biosynthesis in this organism for the first time. Through temperature shift and further fermentation optimization, the crocetin titer reached 30.17 mg/L in shake flasks, surpassing the previous best report in *S. cerevisiae* by 1.51-fold [19].

## Supplementary Information

Additional file 1: Figure S1. Overview of metabolic engineering strategies to improve crocetin production in microorganisms. Figure S2. Strain construction tree in this research. Figure S3. Standard curves for carotenoid quantification by HPLC. Figure S4. Absorbance spectra of supernatants of β-carotene and crocetin-producing strains after fermentation at 30°C for 7 days. Figure S5. Extracellular and intracellular analysis of carotenoids. Figure S6. Culture broth of crocetin producer under different conditions. Figure S7. Analysis of cell death during crocetin fermentation using YLC01 strain. Figure S8. Introducing an exogenous ALD. Figure. S9 Effects of glucose concentration on crocetin contents in *Y. lipolytica* YLC01. Table S1. Biosynthetic genes used in this research. Table S2. Primers used in this research. Table S3. All yeast strains and plasmids constructed in this research. Table S4. Media optimization based on YPD and calculation of C/N ratios.

## Data Availability

No datasets were generated or analyzed during the current study.
