# Supplementary material for: Metabolic engineering of Yarrowia lipolytica for the production and secretion of the saffron ingredient crocetin
Source: Biotechnol Biofuels Bioprod. 2025 Jan 7;18:1. doi: 10.1186/s13068-024-02598-y (PMC11706156; doi:10.1186/s13068-024-02598-y)
Supplement: Supplementary file 1 — Additional file 1: Figure S1. Overview of metabolic engineering strategies to improve crocetin production in microorganisms. Figure S2. Strain construction tree in this research. Figure S3. Standard curves for carotenoid quantification by HPLC. Figure S4. Absorbance spectra of supernatants of β-carotene and crocetin-producing strains after fermentation at 30°C for 7 days. Figure S5. Extracellular and intracellular analysis of carotenoids. Figure S6. Culture broth of crocetin producer under different conditions. Figure S7. Analysis of cell death during crocetin fermentation using YLC01 strain. Figure S8. Introducing an exogenous ALD. Figure. S9 Effects of glucose concentration on crocetin contents in Y. lipolytica YLC01. Table S1. Biosynthetic genes used in this research. Table S2. Primers used in this research. Table S3. All yeast strains and plasmids constructed in this research. Table S4. Media optimization based on YPD and calculation of C/N ratios. [file 13068_2024_2598_MOESM1_ESM.docx]

**Supplementary information**

**Metabolic engineering of *Yarrowia lipolytica* for the production and secretion of the saffron ingredient crocetin**

Tingan Zhou^1, 2^, Young-Kyoung Park^1, 3,*^, Jing Fu^1^, Piotr Hapeta^1^, Cinzia Klemm^1^, Rodrigo Ledesma-Amaro^1,*^

^1^ Department of Bioengineering and Centre for Synthetic Biology, Imperial College London, London SW7 2AZ, UK

^2^ Department of Natural Product Biosynthesis, Max Planck Institute for Chemical Ecology, Jena 07745, Germany

^3^ Université Paris-Saclay, INRAE, AgroParisTech, Micalis Institute, 78350 Jouy-en-Josas, France

^*^ Corresponding author address:

Rodrigo Ledesma-Amaro

Department of Bioengineering and Centre for Synthetic Biology, Imperial College London, London SW7 2AZ, UK. Email address: [r.ledesma-amaro@imperial.ac.uk](mailto:r.ledesma-amaro@imperial.ac.uk)

Young-Kyoung Park

Université Paris-Saclay, INRAE, AgroParisTech, Micalis Institute, 78350 Jouy-en-Josas, France. Email address : youngkyoung.park@inrae.fr

**Supplementary figures**


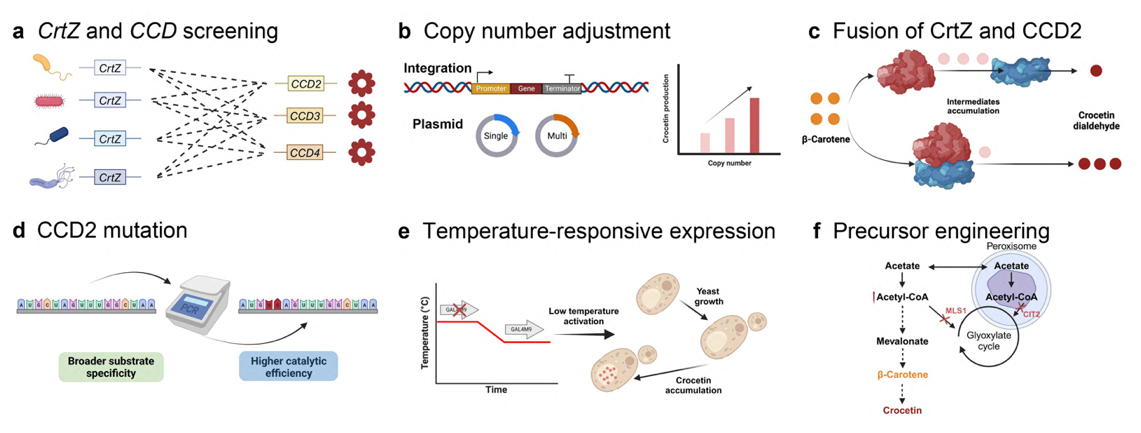


**Figure S1.** Overview of metabolic engineering strategies to improve crocetin production in microorganisms. a, Screening CrtZ and CCD from different sources and having different combinations; b, Adjusting copy number by genome integration or single/multi-copy plasmids; c, Fusion of CrtZ and CCD2 to facilitate catalytic efficiency; d, Mutation of CCD2; e, Construction of GAL4 variant GAL4M9 for temperature response; f, Deletion of the genes involved in glyoxylate cycle to improve acetyl-CoA supplement.

**
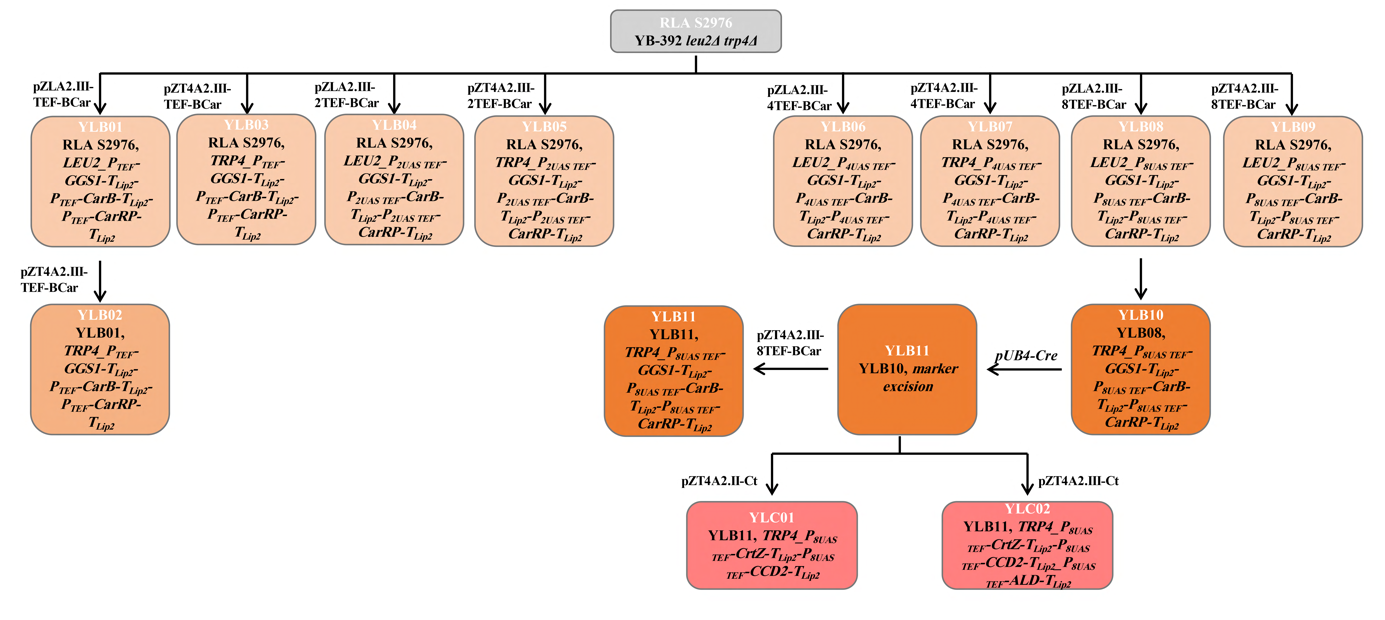
**

**Figure S2.** Strain construction tree in this research.

**
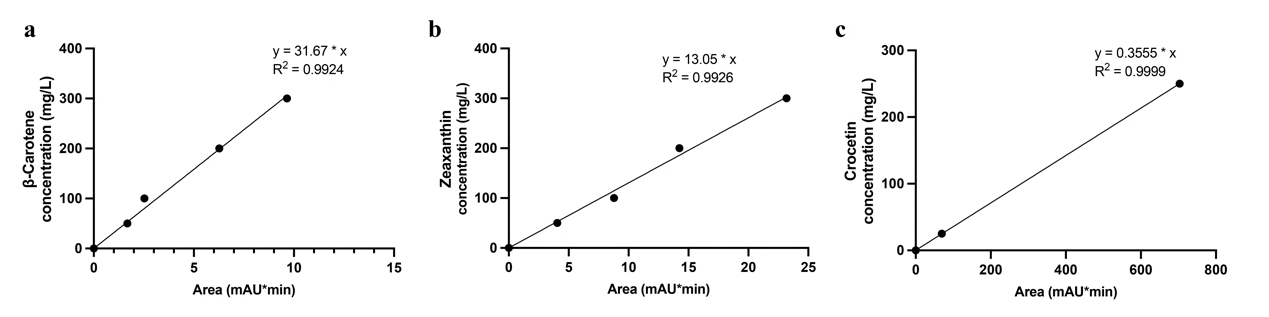
**

**Figure S3.** Standard curves for carotenoid quantification by HPLC. a, β-carotene standard curve using C30 column; b, zeaxanthin standard curve using C30 column; c, crocetin standard curve using C18 column.

**
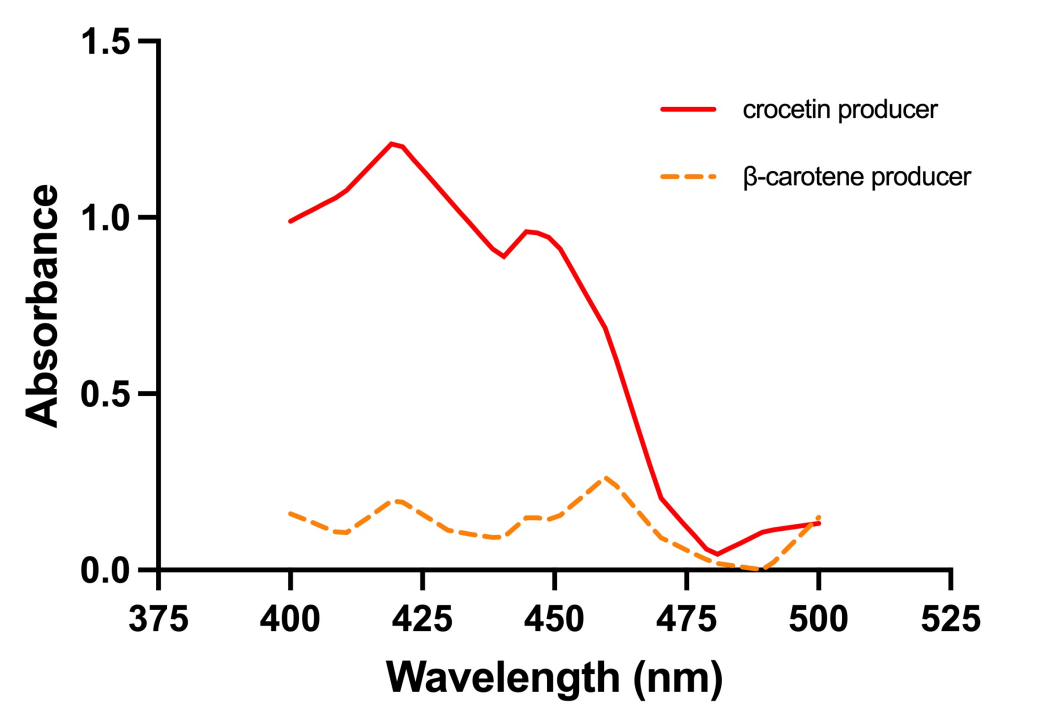
**

**Figure S4.** Absorbance spectra of supernatants of β-carotene and crocetin producer strains after fermentation at 30 °C for 7 days.

**
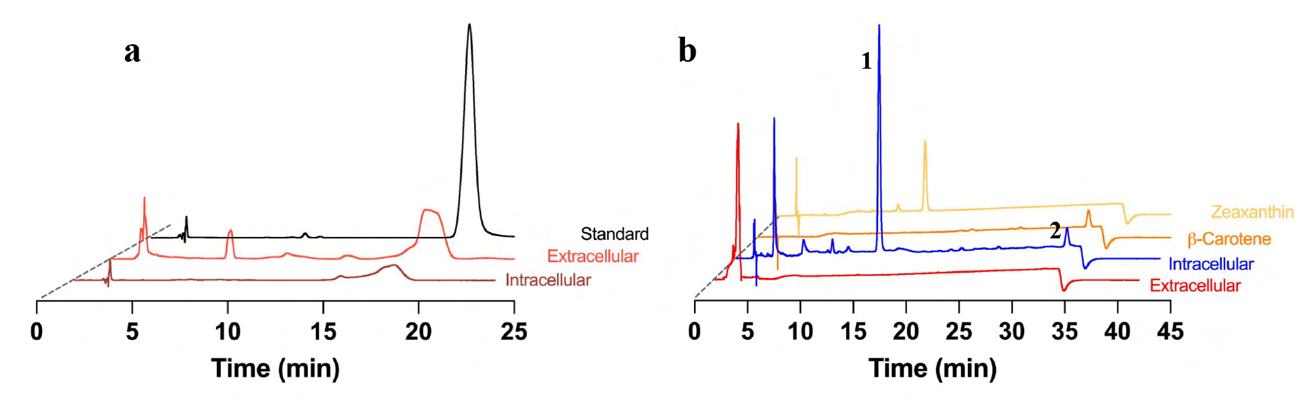
**

**Figure S5.** Extracellular and intracellular analysis of carotenoids. a, HPLC chromatograms of crocetin standard, intracellular and extracellular crocetin biosynthesized by yeast; b, HPLC chromatograms of standards (β-carotene in orange and zeaxanthin in yellow) and carotenoids from the cultivation of YLC01 crocetin-producing strain (extracellular in red and intracellular in blue). Main peak #1 corresponds to zeaxanthin and #2 to β-carotene.


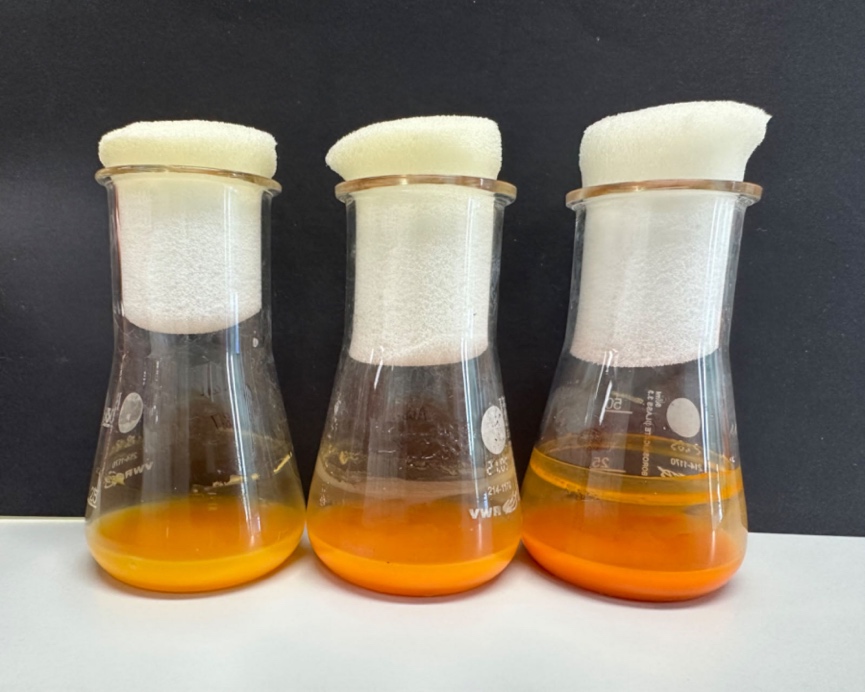


**Figure S6.** Culture broth of crocetin producer YLC01 under different conditions. From left to right: 20 °C, two-step fermentation, 30 °C.

**
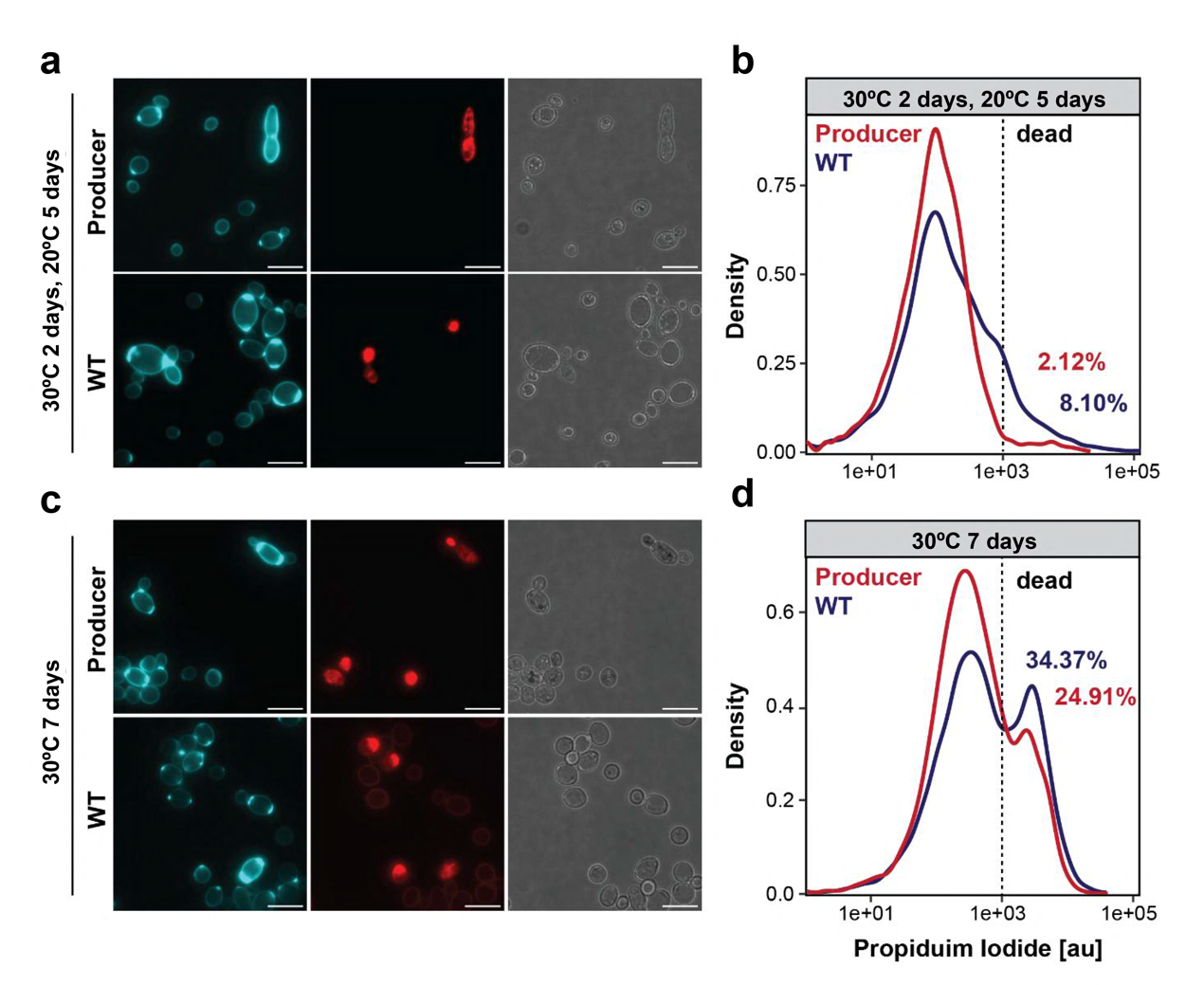
**

**Figure S7.** Analysis of cell death during crocetin fermentation using YLC01 strain. a, c, Microscope images showing crocetin producer and wild-type strains after fermentation at different temperatures. Yeast cells were stained and identified using Calcofluor White (cyan), and Propidium Iodide (red) was used to visualize cell death. Scale bars are 10 μm; b, d, Flow cytometry analysis of cell death during crocetin fermentation at different temperatures. 10,000 Cells were analyzed per condition and strain, showing decreased cell death after incubation using two-step temperature shift fermentation compared to 7 days at 30 ºC.


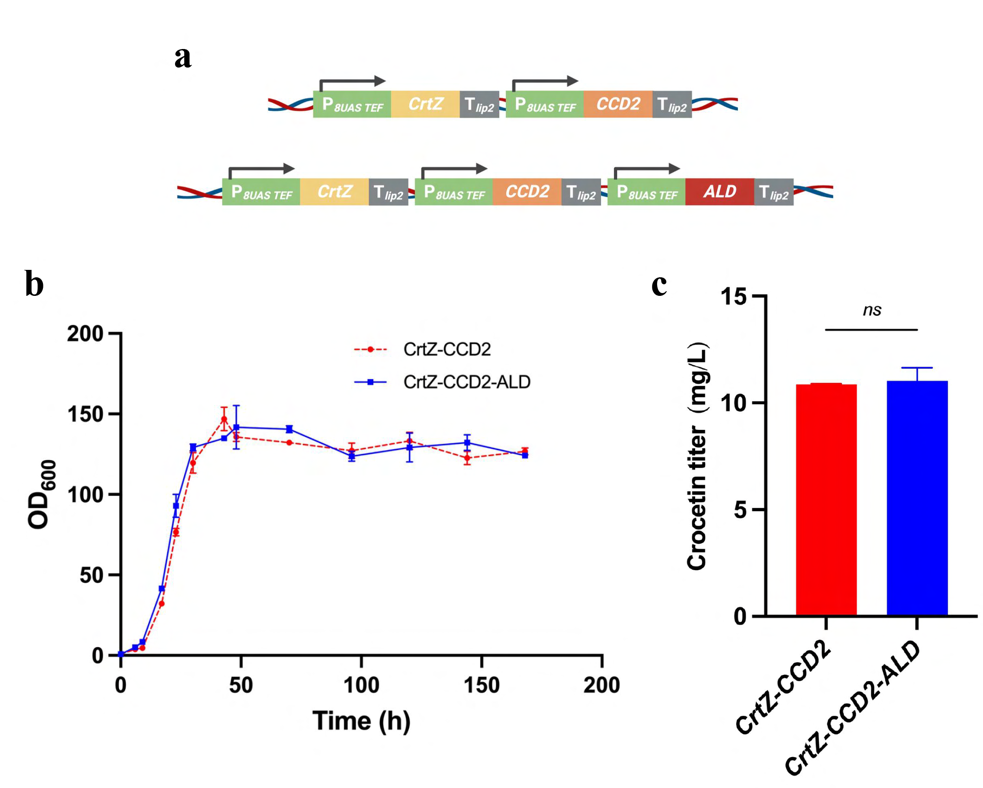


**Figure S8.** Introducing an exogenous ALD. a, Construction of *CrtZ-CCD2-ALD* crocetin cassette; b, Influence of exogenous ALD on yeast growth; c, Influence of exogenous ALD on crocetin titer. YLC01 and YLC02 strains were cultivated using the two-step fermentation method (2 days at 30 °C and then 5 days at 20 °C).

**
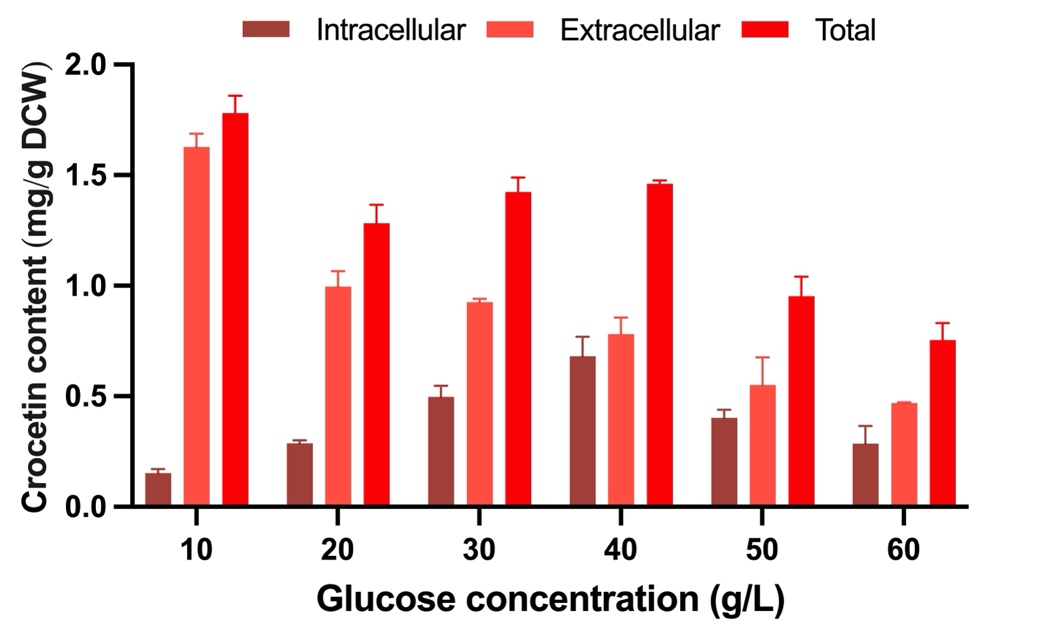
**

**Figure S9.** Effects of glucose concentration on crocetin contents in *Y. lipolytica* YLC01. YLC01 was cultivated using the two-step fermentation method (2 days at 30 °C and then 5 days at 20 °C) when optimizing the glucose concentration.

**Supplementary tables**

**Table S1.** Biosynthetic genes used in this research

| **Gene** | **Sequence** |
| --- | --- |
| *GGS1* | ATGGATTATAACAGCGCGGATTTCAAGGAGATATGGGGCAAGGCCGCCGACACCGCGCTGCTGGGACCGTACAACTACCTCGCCAACAACCGGGGCCACAACATCAGAGAACACTTGATCGCAGCGTTCGGAGCGGTTATCAAGGTGGACAAGAGCGATCTCGAGACTATTTCGCACATCACCAAGATTTTGCATAACTCGTCGCTGCTTGTTGATGACGTGGAAGACAACTCGATGCTCCGACGAGGCCTGCCGGCAGCCCATTGTCTGTTTGGAGTCCCCCAAACCATCAACTCCGCCAACTACATGTACTTTGTGGCTCTGCAGGAGGTGCTCAAGCTCAAGTCTTATGATGCCGTGTCCATTTTCACCGAGGAAATGATCAACTTGCATAGAGGTCAGGGTATGGATCTCTACTGGAGAGAAACACTCACTTGCCCCTCGGAAGACGAGTATCTGGAGATGGTGGTGCACAAGACCGGTGGACTGTTTCGGCTGGCTCTGAGACTTATGCTGTCGGTGGCATCGAAACAGGAGGACCATGAAAAGATCAACTTTGATCTCACACACCTTACCGACACACTGGGAGTCATTTACCAGATTCTGGATGATTACCTCAACCTGCAGTCCACGGAATTGACCGAGAACAAGGGATTCTGCGAAGATATCAGCGAAGGAAAGTTTTCGTTTCCGCTGATTCACAGCATACGCACCAACCCGGATAACCACGAGATTCTCAACATTCTCAAACAGCGAACAAGCGACGCTTCACTCAAAAAGTACGCCGTGGACTACATGAGAACAGAAACCAAGAGTTTCGACTACTGCCTCAAGAGGATACAGGCCATGTCACTCAAGGCAAGTTCGTACATTGATGATCTAGCAGCAGCTGGCCACGATGTCTCCAAGCTACGAGCCATTTTGCATTATTTTGTGTCCACCTCTGACTGTGAGGAGAGAAAGTACTTTGAGGATGCGCAGTGA |
| *CarB* | ATGTCCAAGAAACACATTGTCATTATCGGTGCTGGCGTGGGTGGCACGGCTACAGCTGCTCGTTTGGCCCGCGAAGGCTTCAAGGTCACTGTGGTGGAGAAAAACGACTTTGGTGGCGGCCGATGCTCCTTGATCCATCACCAGGGCCATCGCTTTGATCAGGGCCCGTCGCTCTACCTGATGCCCAAGTACTTTGAGGACGCCTTTGCCGATCTGGACGAGCGCATTCAAGACCACCTGGAGCTGCTGCGATGCGACAACAACTACAAGGTGCACTTTGACGACGGTGAGTCGATCCAGCTGTCGTCTGACTTGACACGCATGAAGGCTGAATTGGACCGCGTGGAGGGCCCCCTTGGTTTTGGCCGATTCCTGGATTTCATGAAAGAGACACACATCCACTACGAAAGCGGCACCCTGATTGCGCTCAAGAAGAATTTCGAATCCATCTGGGACCTGATTCGCATCAAGTACGCTCCAGAGATCTTTCGCTTGCACCTGTTTGGCAAGATCTACGACCGCGCTTCCAAGTACTTCAAGACCAAGAAGATGCGCATGGCATTCACGTTTCAGACCATGTATATGGGCATGTCGCCCTACGATGCGCCTGCTGTCTACAGCCTGTTGCAGTACACCGAGTTCGCTGAAGGCATCTGGTATCCCCGTGGCGGCTTCAACATGGTGGTTCAGAAGCTAGAGGCGATTGCAAAGCAAAAGTACGATGCCGAGTTTATCTACAATGCGCCTGTTGCCAAGATTAACACCGATGATGCCACCAAACAAGTGACAGGTGTAACCTTGGAAAATGGCCACATCATCGATGCCGATGCGGTTGTGTGTAACGCAGATCTGGTCTATGCTTATCACAATCTGTTGCCTCCCTGCCGATGGACGCAAAACACACTGGCTTCCAAGAAATTGACGTCTTCTTCCATTTCCTTCTACTGGTCCATGTCCACCAAGGTGCCTCAATTGGACGTGCACAACATCTTTTTGGCCGAGGCTTATCAGGAGAGCTTTGACGAAATCTTCAAGGACTTTGGCCTGCCTTCTGAAGCCTCCTTCTACGTCAATGTGCCCTCTCGCATCGATCCTTCTGCTGCTCCCGACGGCAAGGACTCTGTCATTGTCTTGGTGCCTATTGGTCATATGAAGAGCAAGACGGGCGATGCTTCCACCGAGAACTACCCGGCCATGGTGGACAAGGCACGCAAGATGGTGCTGGCTGTGATTGAGCGTCGTCTGGGCATGTCGAATTTCGCCGACTTGATTGAGCATGAGCAAGTCAATGATCCCGCTGTATGGCAGAGCAAGTTCAATCTGTGGAGAGGCTCAATTCTGGGTTTGTCTCATGATGTGCTTCAGGTGCTGTGGTTCCGTCCCAGCACAAAGGATTCTACCGGTCGTTATGATAACCTATTCTTTGTGGGTGCAAGCACGCATCCCGGAACTGGTGTTCCCATTGTCCTTGCAGGAAGCAAGCTCACCTCTGACCAAGTTGTCAAGAGCTTTGGAAAGACGCCCAAGCCAAGAAAGATCGAGATGGAGAACACGCAAGCACCTTTGGAGGAGCCTGATGCTGAATCGACATTCCCTGTGTGGTTCTGGTTGCGCGCTGCCTTTTGGGTCATGTTTATGTTCTTTTACTTCTTCCCTCAATCCAATGGCCAAACGCCCGCATCTTTTATCAATAATTTGTTACCTGAAGTATTCCGCGTTCATAACTCTAATGTCATTTAA |
| *CarRP* | ATGCTGCTCACCTACATGGAAGTCCACCTCTACTACACGCTGCCTGTGCTGGGCGTCCTGTCCTGGCTGTCGCGGCCGTACTACACAGCCACCGATGCGCTCAAATTCAAATTTCTGACACTGGTTGCCTTCACGACCGCCTCCGCCTGGGACAACTACATTGTCTACCACAAGGCGTGGTCCTACTGCCCCACCTGCGTCACCGCTGTCATTGGCTACGTGCCCTTGGAGGAGTACATGTTCTTCATCATCATGACTCTGTTGACCGTGGCATTCACCAATCTGGTGATGCGCTGGCACCTGCACAGCTTCTTTATCAGGCCTGAAACGCCCGTCATGCAGTCCGTCCTGGTCCGTCTTGTCCCCATAACAGCCTTATTAATCACTGCATACAAGGCTTGGCATTTGGCGGTCCCTGGAAAGCCACTGTTCTACGGATCATGCATTTTGTGGTACGCCTGTCCGGTTTTGGCCTTATTGTGGTTTGGTGCTGGCGAGTACATGATGCGTCGTCCGCTGGCGGTGCTCGTGTCCATTGCGCTGCCCACGCTGTTTCTCTGCTGGGTCGATGTCGTCGCTATTGGCGCCGGCACATGGGACATTTCGCTGGCCACAAGCACCGGCAAGTTCGTCGTGCCCCACCTGCCCGTGGAGGAATTCATGTTCTTTGCGCTAATTAATACCGTTTTGGTATTTGGTACGTGTGCGATCGATCGCACGATGGCGATCCTCCACCTGTTCAAAAACAAGAGTCCTTATCAGCGCCCATACCAGCACAGCAAGTCGTTCCTCCACCAGATCCTCGAGATGACCTGGGCCTTCTGTTTACCCGACCAAGTGCTGCATTCAGACACATTCCACGACCTGTCCGTCAGCTGGGACATCCTGCGCAAGGCCTCCAAGTCCTTTTACACGGCCTCTGCTGTCTTTCCCGGCGACGTGCGCCAAGAGCTCGGTGTGCTATACGCCTTTTGCAGAGCCACGGACGATCTCTGCGACAACGAGCAGGTCCCTGTGCAGACGCGAAAGGAGCAGCTGATACTGACACATCAGTTCGTCAGCGATCTGTTTGGCCAAAAGACAAGCGCGCCGACTGCCATTGACTGGGACTTTTACAACGACCAACTGCCTGCCTCGTGCATCTCTGCCTTCAAGTCGTTCACCCGTTTGCGCCATGTGCTGGAAGCTGGAGCCATCAAGGAACTGCTCGACGGGTACAAGTGGGATTTGGAGCGTCGCTCCATCAGGGATCAGGAGGATCTCAGATATTACTCAGCTTGTGTCGCCAGCAGTGTTGGTGAAATGTGCACTCGCATCATACTGGCCCACGCCGACAAGCCCGCCTCCCGCCAGCAAACACAGTGGATCATTCAGCGTGCGCGTGAAATGGGTCTGGTACTCCAATATACAAACATTGCAAGAGACATTGTCACCGACAGCGAGGAACTGGGCAGATGCTACCTGCCTCAGGATTGGCTTACCGAGAAGGAGGTGGCGCTGATTCAAGGCGGCCTTGCCCGAGAAATTGGCGAGGAGCGATTGCTCTCACTGTCGCATCGCCTCATCTACCAGGCAGACGAGCTCATGGTGGTTGCCAACAAGGGCATCGACAAGCTGCCCAGCCATTGTCAAGGCGGCGTGCGTGCGGCCTGCAACGTCTATGCTTCCATTGGCACCAAGCTCAAGTCTTACAAGCACCACTATCCCAGCAGAGCACATGTCGGCAATTCGAAACGAGTGGAAATTGCTCTTCTTAGCGTATACAACCTTTACACCGCGCCAATTGCGACTAGTAGTACCACACATTGCAGACAGGGAAAAATGAGAAATCTAAATACCATTTAA |
| *CrtZ* | ATGCTGTGGATCTGGAACGCCCTGATCGTGTTCGTGACCGTGGTCGGCATGGAAGTGGTGGCCGCTCTGGCCCACAAGTACATCATGCACGGCTGGGGCTGGGGATGGCACCTGTCTCACCACGAGCCTCGAAAGGGCGCCTTCGAGGTGAACGACCTGTACGCCGTGGTGTTCGCCATCGTGTCTATTGCCCTGATCTACTTCGGCTCTACCGGCATCTGGCCCCTGCAGTGGATCGGAGCCGGCATGACCGCCTACGGCCTGCTGTACTTCATGGTGCACGACGGCCTGGTCCACCAGAGATGGCCCTTCCGGTACATTCCCCGAAAGGGCTACCTGAAGCGACTGTACATGGCCCACCGAATGCACCACGCCGTGCGAGGCAAGGAAGGCTGCGTTTCTTTCGGCTTCCTGTACGCACCTCCTCTGTCTAAGCTGCAGGCTACCCTGCGAGAGCGACACGCCGCTCGATCTGGCGCTGCCCGAGATGAGCAGGACGGCGTGGACACCTCTTCGTCTGGCAAGTAA |
| *CCD2* S323A | ATGGCCAACAAGGAAGAGGCCGAGAAGCGAAAGAAGAAGCCCAAGCCTCTGAAGGTGCTGATCACCAAGGTGGACCCTAAGCCTCGAAAGGGCATGGCCTCTGTGGCCGTGGACCTGCTCGAGAAGGCTTTCGTGTACCTGCTGTCTGGCAACTCTGCCGCCGACCGATCTTCTTCTTCTGGCCGACGACGACGAAAGGAACACTACTACCTGTCTGGAAACTACGCTCCCGTGGGCCACGAGACTCCTCCATCTGACCATCTGCCTATCCACGGATCTCTGCCCGAGTGCCTGAACGGCGTGTTCCTGCGAGTGGGCCCCAACCCTAAGTTCGCCCCTGTGGCCGGCTACAACTGGGTGGACGGCGACGGCATGATCCACGGCCTGCGAATCAAGGACGGCAAGGCCACCTACCTGTCGCGGTACATCAAGACCTCTCGATTCAAGCAAGAGGAATACTTCGGCCGAGCCAAGTTCATGAAGATCGGCGACCTGCGAGGCCTGCTGGGATTCTTCACCATCCTGATCCTGGTGCTGCGAACTACCCTGAAGGTGATCGACATCTCTTACGGCCGAGGCACCGGCAACACCGCTCTGGTGTACCACAACGGCCTGCTGCTGGCCCTGTCTGAAGAGGACAAGCCCTACGTGGTGAAGGTTCTCGAGGACGGTGACCTGCAGACCCTGGGCATCCTGGACTACGACAAGAAGCTGTCTCACCCCTTCACCGCTCATCCCAAGATCGACCCTCTGACCGACGAGATGTTCACCTTCGGCTACTCTATCTCTCCTCCTTACCTGACCTACCGAGTGATCTCTAAGGACGGCGTGATGCAGGACCCCGTGCAGATCTCTATCACCTCTCCTACCATCATGCACGACTTCGCCATCACCGAGAACTACGCCATTTTCATGGACCTGCCTCTGTACTTCCAGCCTGAGGAAATGGTGAAGGGCAAGTTCGTGGCCTCTTTTCACCCCACCAAGCGAGCCCGAATCGGCGTGCTGCCCCGATACGCCAAGGACGAGCACCCCATCCGATGGTTCGACCTGCCTTCTTGCTTCATGACCCACAACGCCAACGCCTGGGAAGAGAACGACGAGGTGGTGCTGTTCACCTGTCGACTGGAATCTCCCGACCTGGACATGCTGTCGGGACCCGCCGAGGAAGAGATCGGCAACTCTAAGTCTGAGCTGTACGAGATGCGATTCAACCTCAAGACCGGCATTACCTCTCAAAAGCAGCTGTCTGTGCCCTCTGTGGACTTCCCTCGAATCAACCAGTCTTACACCGGCCGAAAGCAGCAGTACGTGTACTGCACCCTGGGAAACACCAAGATCAAGGGCATCGTGAAGTTCGACCTCCAGATCGAGCCCGAGGCCGGCAAGACCATGCTCGAGGTCGGCGGAAACGTGCAGGGCATCTTCGAGCTGGGACCCCGACGATACGGCTCTGAGGCCATCTTCGTGCCCTGTCAGCCCGGCATCAAGTCTGACGAGGACGACGGCTACCTGATTTTCTTCGTCCACGACGAGAACAACGGCAAGTCTGAGGTGAACGTGATTGACGCTAAGACCATGTCTGCTGAGCCCGTGGCCGTCGTCGAGCTGCCCTCTCGAGTGCCCTACGGCTTCCACGCTCTGTTTCTGAACGAGGAAGAACTGCAGAAGCACCAGGCCGAGACTTAA |
| *ALD* | ATGAACACCGCCAAGACCGTGGTGGCCGAGCAGCGAGACTTCTTCCGACAGGGCAAGACCAAGTCTGTGCAGGACCGACTGACCGCTCTGGCCAAGCTCAAGACCCAGATTCAGGCCCAAGAGGAAGAGATCATCAAGGCCCTGAAGCAGGACTTCGGCAAGCCCACCTTCGAGTCTTACGTGAACGAGATCCTGGGCGTGATCCGAGAGATCAACTACTACCAAAAGCACCTCCAGCAGTGGTCTAAGCCCCAGCGAGTGGGCACCAACCTGATGGTGTTCCCCGCCTCTGCTCAGCTGCGACCCGAGCCTCTGGGCGTCGTGCTGATCATCTCTCCCTGGAACTACCCCTTCTACCTGTGTCTGATGCCCCTGATCGGCGCCATTGCCGCCGGAAACTGCGTGGTGGTGAAGCCCTCTGAGTACACCCCTGCCATCTCTGGCGTGATCACCCGACTGATCCAGAACGTGTTCTCTCCCGCCTGGGCCACCGTGGTCGAGGGCGACGAGACTATCTCTCAGCAGCTCCTGCAAGAGAAGTTCGACCACATCTTCTTCACCGGCTCTCCCCGAGTGGGACGACTGATCATGGCCGCTGCCGCTGAGCAGCTGACCCCTGTGACTCTGGAACTCGGCGGCAAGTCTCCCTGTGTGGTGGACCGAGAGATTAACCTCCAAGAGACTGCCAAGCGAATCATGTGGGGCAAGCTGGTGAACGCCGGCCAGACCTGCGTGGCTCCCGACTACCTGCTGGTCGAGCAGTCTTGCCTGGAACAGCTGCTGCCCGCTCTGCAGCAGGCCATCCAGATGCTGTTCGGCGAGAACCCCGCTCACTCTCCTGACTACACCCGAATCGTGAACCAGCAGCAATGGTCCCGACTGGTGTCTCTGCTGTCTCACGGCAAGGTGATTACCCGAGGCGACCACAACGAGGGTGACCGATATATTGCTCCCACTCTGATCATCGACCCCGACCTGAACTCGCCCCTGATGCAAGAGGAAATCTTCGGCCCCATTCTGCCCATCCTGACCTACCAGTCTCTGTCTGAGGCCATCGACTTCATCAACATCAAGCCCAAGCCTCTGGCTCTGTACTTCTTCTCGAACAACCGACAGAAGCAAGAAGAGATCCTGCAGTCTACCTCTTCTGGATCTGTGTGCCTGAACGACATTCTGCTGCACCTGACCGTGACCGACCTGCCTTTCGGCGGCGTGGGCGAGTCTGGCATGGGCCGATACCACGGAAAGGCTACCTTCGACACCCTGTCTAACTACAAGTCTATCCTGCGACGACCCTTCTGGGGCGAGACTAACCTGCGATACTCTCCCTACGGCAAGAAGATGAACCTGATCAAGAAGCTGTTCTCCTAG |
| *P_TEF_* | ACCGGGTTGGCGGCGTATTTGTGTCCCAAAAAACAGCCCCAATTGCCCCAATTGACCCCAAATTGACCCAGTAGCGGGCCCAACCCCGGCGAGAGCCCCCTTCACCCCACATATCAAACCTCCCCCGGTTCCCACACTTGCCGTTAAGGGCGTAGGGTACTGCAGTCTGGAATCTACGCTTGTTCAGACTTTGTACTAGTTTCTTTGTCTGGCCATCCGGGTAACCCATGCCGGACGCAAAATAGACTACTGAAAATTTTTTTGCTTTGTGGTTGGGACTTTAGCCAAGGGTATAAAAGACCACCGTCCCCGAATTACCTTTCCTCTTCTTTTCTCTCTCTCCTTGTCAACTCACACCCGAAATCGTTAAGCATTTCCTTCTGAGTATAAGAATCATTCAA |
| *UAS* | CATGCTGAGGTGTCTCACAAGTGCCGTGCAGTCCCGCCCCCACTTGCTTCTCTTTGTGTGTAGTGTACGTACATTATCGAGAGGGTTGTTCCCGCCCACCTCGATCCGG |
| *T_Lip2_* | GTGTCTGTGGTATCTAAGCTATTTATCACTCTTTACAACTTCTACCTCAACTATCTACTTTAATAAATGAATATCGTTTATTCTCTATGATTACTGTATATGCGTTCCTCTAAGACAAATCG |

**Table S2.** Primers used in this research

| **Primers** | **Sequence** | **Description** |
| --- | --- | --- |
| *TEF-Int-F* | CCATGCCGGACGCAAAATAGACTAC | Colony PCR verification |
| *GGS1-Int-R* | CACCACCATCTCCAGATACTCG | Colony PCR verification |
| *CrtB-Int-R* | CTGTAGACAGCAGGCGCATC | Colony PCR verification |
| *CrtRP-Int-R* | GAACGACTTGCTGTGCTGG | Colony PCR verification |
| *CrtZ-Int-R* | GGAATGTACCGGAAGGGCCATCTC | Colony PCR verification |
| *CCD2-Int-R* | GGATGGTGAAGAATCCCAGCAGGC | Colony PCR verification |
| *ALD-Int-R* | CCACATGATTCGCTTGGCAGTCTC | Colony PCR verification |

**Table S3.** All yeast strains and plasmids constructed in this research

| **Strain/ Plasmid** | **Description** | **Source** |  |
| --- | --- | --- | --- |
| **Strain** |  |  |  |
| RLA S276 | WT YB-392 | ARS Culture collection |  |
| RLA S2976 | YB-392 *leu2Δ trp4Δ* | This study |  |
| YLB01 | RLA S2976 + LEU2_P*_TEF_*-*GGS1*-T*_Lip2_*-P*_TEF_*-*CarB*-T*_Lip2_*-P*_TEF_*-*CarRP*-T*_Lip2_* | This study |  |
| YLB02 | YLB01 + TRP4_P_TEF_-*GGS1*-T_Lip2_-P_TEF_-*CarB*-T_Lip2_-P_TEF_-*CarRP*-T_Lip2_ | This study |  |
| YLB03 | RLA S2976 + TRP4_P_TEF_-*GGS1*-T_Lip2_-P_TEF_-*CarB*-T_Lip2_-P_TEF_-*CarRP*-T_Lip2_ | This study |  |
| YLB04 | RLA S2976 + LEU2_P*_2UAS TEF_*-*GGS1*-T*_Lip2_*-P*_2UAS TEF_*-*CarB*-T*_Lip2_*-P*_2UAS TEF_*-*CarRP*-T*_Lip2_* | This study |  |
| YLB05 | RLA S2976 + TRP4_P_2UAS TEF_-*GGS1*-T_Lip2_-P_2UAS TEF_-*CarB*-T_Lip2_-P_2UAS TEF_-*CarRP*-T_Lip2_ | This study |  |
| YLB06 | RLA S2976 + LEU2_P*_4UAS TEF_*-*GGS1*-T*_Lip2_*-P*_4UAS TEF_*-*CarB*-T*_Lip2_*-P*_4UAS TEF_*-*CarRP*-T*_Lip2_* | This study |  |
| YLB07 | RLA S2976 + TRP4_P_4UAS TEF_-*GGS1*-T_Lip2_-P_4UAS TEF_-*CarB*-T_Lip2_-P_4UAS TEF_-*CarRP*-T_Lip2_ | This study |  |
| YLB08 | RLA S2976 + LEU2_P_8UAS TEF_-*GGS1*-T_Lip2_-P_8UAS TEF_-*CarB*-T_Lip2_-P_8UAS TEF_-*CarRP*-T_Lip2_ | This study |  |
| YLB09 | RLA S2976 + TRP4_P_8UAS TEF_-*GGS1*-T_Lip2_-P_8UAS TEF_-*CarB*-T_Lip2_-P_8UAS TEF_-*CarRP*-T_Lip2_ | This study |  |
| YLB10 | YLB08 + TRP4_P_8UAS TEF_-*GGS1*-T_Lip2_-P_8UAS TEF_-*CarB*-T_Lip2_-P_8UAS TEF_-*CarRP*-T_Lip2_ | This study |  |
| YLB11 | YLP + P_8UAS TEF_-*GGS1*-T_Lip2_-P_8UAS TEF_-*CarB*-T_Lip2_-P_8UAS TEF_-*CarRP*-T_Lip2_ + P_8UAS TEF_-*GGS1*-T_Lip2_-P_8UAS TEF_-*CarB*-T_Lip2_-P_8UAS TEF_-*CarRP*-T_Lip2_ | This study |  |
| YLB12 | YLB11 + TRP4_P_8UAS TEF_-*GGS1*-T_Lip2_-P_8UAS TEF_-*CarB*-T_Lip2_-P_8UAS TEF_-*CarRP*-T_Lip2_ | This study |  |
| YLC01 | YLB12, + TRP4_P_8UAS TEF_-*CrtZ*-T_Lip2_-P_8UAS TEF_-*CCD2*-T_Lip2_ | This study |  |
| YLC02 | YLB12 + TRP4_P_8UAS TEF_-*CrtZ*-T_Lip2_-P_8UAS TEF_-*CCD2*-T_Lip2_-P_8UAS TEF_-*ALD*-T_Lip2_ | This study |  |
| **Plasmid** |  |  |  |
| pYaTK | Lv0 cloning vector used for storing synthetic genes and basic parts, Chl^r^ marker | Yuzbashev et al. 2023 |  |
| pZUS1.1/2/3 | Lv1 vector used for assembling promoters, genes, and terminators, URA3 and Spec^r^ marker | Yuzbashev et al. 2023 |  |
| pZLA2.III | Lv2 vector used for assembling 3 Lv1 cassettes, LEU2 and Amp^r^ marker | Yuzbashev et al. 2023 |  |
| pZT4A2.II | Lv2 vector used for assembling 2 Lv1 cassettes, TRP4 and Amp^r^ marker | This study |  |
| pZT4A2.III | Lv2 vector used for assembling 3 Lv1 cassettes, TRP4 and Amp^r^ marker | This study |  |
| pYaTK-TEF | Promoter P*_TEF_* cloned into pYaTK | Yuzbashev et al. 2023 |  |
| pYaTK-2UAS TEF | | Promoter P_2UAS TEF_ cloned into pYaTK | Yuzbashev et al. 2023 |
| pYaTK-4UAS TEF | | Promoter P_4UAS TEF_ cloned into pYaTK | Yuzbashev et al. 2023 |
| pYaTK-8UAS TEF | Promoter P_8UAS TEF_ cloned into pYaTK | Yuzbashev et al. 2023 |  |
| pTaTK-Tlip2 | Terminator T_Lip2_ cloned into pYaTK | Yuzbashev et al. 2023 |  |
| \| \| pYaTK-*GGS1* \|  \|  \| \| --- \| --- \| --- \| \|  \|  \| \| --- \| --- \| --- \| --- \| --- \| --- \| | *GGS1* was synthesized after codon optimization and cloned into pYaTK | This study |  |
| \| \| pYaTK-*CarB* \|  \|  \| \| --- \| --- \| --- \| \|  \|  \| \| --- \| --- \| --- \| --- \| --- \| --- \| | *CarB* was synthesized after codon optimization and cloned into pYaTK | This study |  |
| \| \| pYaTK-*CarRP* \|  \|  \| \| --- \| --- \| --- \| \|  \|  \| \| --- \| --- \| --- \| --- \| --- \| --- \| | *CarRP* was synthesized after codon optimization and cloned into pYaTK | This study |  |
| \| \| pYaTK-*CrtZ* \|  \|  \| \| --- \| --- \| --- \| \|  \|  \| \| --- \| --- \| --- \| --- \| --- \| --- \| | *CrtZ* was synthesized after codon optimization and cloned into pYaTK | This study |  |
| \| \| pYaTK-*CCD2* \|  \|  \| \| --- \| --- \| --- \| \|  \|  \| \| --- \| --- \| --- \| --- \| --- \| --- \| | *CCD2 was* synthesized after codon optimization and cloned into pYaTK | This study |  |
| \| \| pYaTK-*ALD* \|  \|  \| \| --- \| --- \| --- \| \|  \|  \| \| --- \| --- \| --- \| --- \| --- \| --- \| | *ALD* was synthesized after codon optimization and cloned into pYaTK | This study |  |
| pZUS1.1-TEF-*GGS1* | P*_TEF_*-*GGS1*-T*_Lip2_* was assembled and inserted into pZUS1.1 | This study |  |
| pZUS1.2-TEF -*CarB* | P*_TEF_*-*CarB*-T*_Lip2_* was assembled and inserted into pZUS1.2 | This study |  |
| pZUS1.3-TEF-*CarRP* | P*_TEF_*-*CarRP*-T*_Lip2_* was assembled and inserted into pZUS1.3 | This study |  |
| pZUS1.1-2TEF-*GGS1* | | P_2UAS TEF_-*GGS1*-T*_Lip2_* was assembled and inserted into pZUS1.1 | This study |
| pZUS1.2-2TEF-*CarB* | | P_2UAS TEF_-*CarB*-T*_Lip2_* was assembled and inserted into pZUS1.2 | This study |
| pZUS1.3-2TEF-*CarRP* | | P_2UAS TEF_-*CarRP*-T*_Lip2_* was assembled and inserted into pZUS1.3 | This study |
| pZUS1.1-4TEF-*GGS1* | | P_2UAS TEF_-*GGS1*-T*_Lip2_* was assembled and inserted into pZUS1.1 | This study |
| pZUS1.2-4TEF-*CarB* | | P_2UAS TEF_-*CarB*-T*_Lip2_* was assembled and inserted into pZUS1.2 | This study |
| pZUS1.3-4TEF-*CarRP* | | P_4UAS TEF_-*CarRP*-T*_Lip2_* was assembled and inserted into pZUS1.3 | This study |
| pZUS1.1-8TEF-*GGS1* | P_8UAS TEF_-*GGS1*-T*_Lip2_* was assembled and inserted into pZUS1.1 | This study |  |
| pZUS1.2-8TEF-*CarB* | P_8UAS TEF_-*CarB*-T*_Lip2_* was assembled and inserted into pZUS1.2 | This study |  |
| pZUS1.3-8TEF-*CarRP* | P_8UAS TEF_-*CarRP*-T*_Lip2_* was assembled and inserted into pZUS1.3 | This study |  |
| pZUS1.1-8TEF-*CrtZ* | P_8UAS TEF_-*CrtZ*-T*_Lip2_* was assembled and inserted into pZUS1.1 | This study |  |
| pZUS1.2-8TEF-*CCD2* | P_8UAS TEF_-*CCD2*-T*_Lip2_* was assembled and inserted into pZUS1.2 | This study |  |
| pZUS1.3-8TEF-*ALD* | P_8UAS TEF_-*ALD*-T*_Lip2_* was assembled and inserted into pZUS1.3 | This study |  |
| pZLA2.III-TEF-BCar | pZUS1.1-TEF-*GGS1*, pZUS1.2-TEF–*CarB*, and pZUS1.3-TEF-*CarRP* were assembled together and inserted into pZLA2.III | This study |  |
| pZT4A2.III-TEF-BCar | pZUS1.1-TEF-*GGS1*, pZUS1.2-TEF–*CarB*, and pZUS1.3-TEF-*CarRP* were assembled together and inserted into pZT4A2.III | This study |  |
| pZLA2.III-2TEF-BCar | | pZUS1.1-2TEF-*GGS1*, pZUS1.2-2TEF–*CarB*, and pZUS1.3-2TEF-*CarRP* were assembled together and inserted into pZLA2.III | This study |
| pZT4A2.III-2TEF-BCar | | pZUS1.1-2TEF-*GGS1*, pZUS1.2-2TEF–*CarB*, and pZUS1.3-2TEF-*CarRP* were assembled together and inserted into pZT4A2.III | This study |
| pZLA2.III-4TEF-BCar | | pZUS1.1-4TEF-*GGS1*, pZUS1.2-4TEF–*CarB*, and pZUS1.3-4TEF-*CarRP* were assembled together and inserted into pZLA2.III | This study |
| pZT4A2.III-4TEF-BCar | | pZUS1.1-4TEF-*GGS1*, pZUS1.2-4TEF–*CarB*, and pZUS1.3-4TEF-*CarRP* were assembled together and inserted into pZT4A2.III | This study |
| pZLA2.III-8TEF-BCar | pZUS1.1-8TEF-*GGS1*, pZUS1.2-8TEF–*CarB*, and pZUS1.3-8TEF-*CarRP* were assembled together and inserted into pZLA2.III | This study |  |
| pZT4A2.III-8TEF-BCar | pZUS1.1-8TEF-*GGS1*, pZUS1.2-8TEF–*CarB*, and pZUS1.3-8TEF-*CarRP* were assembled together and inserted into pZT4A2.III | This study |  |
| pZT4A2.II-Ct | pZUS1.1-8TEF-*CrtZ* and pZUS1.2-8TEF-*CCD2* were assembled together and inserted into pZT4A2.II | This study |  |
| pZT4A2.III-Ct | pZUS1.1-8TEF-*CrtZ*, pZUS1.2-8TEF-*CCD2*, and pZUS1.3-8TEF-*ALD* were assembled together and inserted into pZT4A2.III | This study |  |
| pUB4-Cre | Used for yeast marker recovery, Hygro^r^ marker | Fickers et al. 2003 |  |

ReferencesYuzbashev TV, Yuzbasheva EY, Melkina OE, Patel D, Bubnov D, Dietz H, Ledesma-Amaro R. A DNA assembly toolkit to unlock the CRISPR/Cas9 potential for metabolic engineering. Commun Biol. 2023;6(1):858.

Fickers P, Le Dall MT, Gaillardin C, Thonart P, Nicaud JM. New disruption cassettes for rapid gene disruption and marker rescue in the yeast *Yarrowia lipolytica*. J Microbiol Meth. 2003;55(3):727-37

**Table S4.** Media optimization based on YPD and calculation of C/N ratios

| **Medium** | **Glucose concentration (g/L)** | | **Yeast Extract concentration (g/L)** | **Peptone concentration (g/L)** | **C/N ratio** |
| --- | --- | --- | --- | --- | --- |
| Y_5_P_10_D_20_ | 20 | 5 | | 10 | 5:1 |
| Y_10_P_20_D_20_ | 20 | 10 | | 20 | 3:1 |
| Y_15_P_30_D_20_ | 20 | 15 | | 30 | 2:1 |
| Y_20_P_40_D_20_ | 20 | 20 | | 40 | 1:1 |
| Y_2.5_P_5_D_10_ | 10 | 2.5 | | 5 | 5:1 |
| Y_7.5_P_15_D_30_ | 30 | 7.5 | | 15 | 5:1 |
| Y_10_P_20_D_40_ | 40 | 10 | | 20 | 5:1 |
| Y_12.5_P_25_D_50_ | 50 | 12.5 | | 25 | 5:1 |
| Y_15_P_30_D_60_ | 60 | 15 | | 30 | 5:1 |

The C/N ratio was calculated as the molar ratio of carbon and nitrogen. The nitrogen concentration was referred to the manufacturer’s information, and C/N ratio was a rough calculation.

Yeast Extract: 10-12% N (<https://www.sigmaaldrich.com/specification-sheets/338/256/Y0875-BULK.pdf>)

Peptone: 12% N (<https://www.sigmaaldrich.com/GB/en/product/sial/70169>)
